# PtrA Is Functionally Intertwined with GacS in Regulating the Biocontrol Activity of *Pseudomonas chlororaphis* PA23

**DOI:** 10.3389/fmicb.2016.01512

**Published:** 2016-09-22

**Authors:** Nidhi Shah, Natasha Klaponski, Carrie Selin, Rachel Rudney, W. G. Dilantha Fernando, Mark F. Belmonte, Teresa R. de Kievit

**Affiliations:** ^1^Department of Microbiology, University of ManitobaWinnipeg, MB, Canada; ^2^Department of Plant Science, University of ManitobaWinnipeg, MB, Canada; ^3^Department of Biological Science, University of ManitobaWinnipeg, MB, Canada

**Keywords:** antifungal, pyrrolnitrin, phenazine, degradative enzymes, autoinducer

## Abstract

*In vitro* inhibition of the fungal pathogen *Sclerotinia sclerotiorum* by *Pseudomonas chlororaphis* PA23 is reliant upon a LysR-type transcriptional regulator (LTTR) called PtrA. In the current study, we show that Sclerotinia stem rot and leaf infection are significantly increased in canola plants inoculated with the *ptrA*-mutant compared to the wild type, establishing PtrA as an essential regulator of PA23 biocontrol. LTTRs typically regulate targets that are upstream of and divergently transcribed from the LTTR locus. We identified a short chain dehydrogenase (*scd*) gene immediately upstream of *ptrA*. Characterization of a *scd* mutant revealed that it is phenotypically identical to the wild type. Moreover, *scd* transcript abundance was unchanged in the *ptrA* mutant. These findings indicate that PtrA regulation does not involve *scd*, rather this LTTR controls genes located elsewhere on the chromosome. Employing a combination of complementation and transcriptional analysis we investigated whether connections exist between PtrA and other regulators of biocontrol. Besides *ptrA, gacS* was the only gene able to partially rescue the wild-type phenotype, establishing a connection between PtrA and the sensor kinase GacS. Transcriptomic analysis revealed decreased expression of biosynthetic (*phzA, prnA*) and regulatory genes (*phzI, phzR, rpoS, gacA, rsmX, rsmZ, retS*) in the *ptrA* mutant; conversely, *rsmE*, and *rsmY* were markedly upregulated. The transcript abundance of *ptrA* was nine-fold higher in the mutant background indicating that this LTTR negatively autoregulates itself. In summary, PtrA is an essential regulator of genes required for PA23 biocontrol that is functionally intertwined with GacS.

## Introduction

Public concern over the use of chemical pesticides together with the potential for acquiring resistance to these compounds has led to renewed interest in alternative strategies for management of diseases affecting plants. *Pseudomonas chlororaphis* strain PA23 is a soybean root-tip isolate that demonstrates excellent antifungal (AF) activity against *Sclerotinia sclerotiorum* (Lib.) de Bary (Savchuk and Fernando, 2004). Strain PA23 produces an arsenal of compounds including the diffusible antibiotics phenazine 1-carboxylic acid (PCA), 2-hydroxyphenazine (2-OH-PHZ), and pyrrolnitrin (PRN) together with degradative enzymes (Poritsanos et al., 2006; Zhang et al., 2006; Selin et al., 2010). PRN is the primary antibiotic responsible for biocontrol; conversely, phenazines (PHZ) are not essential for fungal suppression, but do play a role in biofilm formation (Selin et al., 2010). Despite the fact that many biocontrol agents perform well in the greenhouse, they often exhibit reduced efficacy under field conditions (Cook, 1993; Walsh et al., 2001; Haas and Keel, 2003). Variable expression of genes and gene products required for biocontrol likely contributes to poor performance in the field. It is essential, therefore, that molecular mechanisms underlying biocontrol are well understood so that production of the pathogen-suppressive factors can be optimized in the environment.

In both pathogenic and biocontrol pseudomonads, expression of secondary metabolites is controlled by a multi-tiered network of regulation. Situated at the top of this hierarchy is the GacS/GacA two-component signal transduction system, comprised of the sensor kinase GacS and its cognate response regulator GacA (Heeb and Haas, 2001). For many biocontrol strains, including PA23, a mutation in *gacS* or *gacA* leads to a loss of fungal antagonism (Heeb and Haas, 2001; Poritsanos et al., 2006). A second system, called Rsm, functions in concert with Gac and consists of a combination of RsmA-like repressor proteins and untranslated regulatory RNAs. The repressor proteins act at the post-transcriptional level by binding to and blocking the ribosome-binding site (RBS) of target mRNA (Lapouge et al., 2008). The regulatory RNAs antagonize repression by titrating out the RsmA-like proteins, rendering RBSs accessible to the translational machinery (Lapouge et al., 2008). Several additional regulatory components govern expression of PA23 AF metabolites including the PhzR/PhzI quorum-sensing (QS) system (Selin et al., 2012), the stationary phase sigma factor RpoS (Manuel et al., 2012), a regulator of RpoS called PsrA (Selin et al., 2014), and the stringent response (Manuel et al., 2012). Cross-regulation between the regulators themselves adds to the increasingly complex nature of this regulatory hierarchy (Manuel et al., 2012; Selin et al., 2012, 2014).

We have recently identified a novel regulator in PA23 called PtrA (Pseudomonas transcriptional regulator) (Klaponski et al., 2014). The phenotype of a *ptrA* mutant is very similar to that of a *gac*-deficient strain exhibiting a complete loss of AF activity (Poritsanos et al., 2006; Selin et al., 2014). PtrA belongs to the LysR-type transcriptional regulator (LTTR) family, which is the most abundant class of transcriptional regulators found among prokaryotes (Schell, 1993). LTTRs act as either activators or repressors and are known to control a diverse range of metabolic functions including cell invasion and virulence, QS, oxidative stress, and amino acid metabolism (Cao et al., 2001; Sperandio et al., 2002; Kim et al., 2004; Heroven and Dersch, 2006; Byrne et al., 2007; Kovaleva and Gelfand, 2007; Hernández-Lucas et al., 2008; Maddocks and Oyston, 2008). Preliminary proteomic and phenotypic analysis of a *ptrA* mutant revealed 59 differentially expressed proteins together with decreased PHZ and PRN production, consistent with the loss of AF activity (Klaponski et al., 2014).

LTTRs frequently control expression of genes that are upstream of and divergently transcribed from the *lttr* locus (Schell, 1993; Maddocks and Oyston, 2008). Immediately upstream of *ptrA* lies a gene encoding a short-chain dehydrogenase, designated *scd*. At present, it is not known what role *scd* plays in PA23 biological control. Moreover connections between PtrA and other members of the regulatory network have not been investigated. The aim of the current study was to conduct greenhouse studies to establish whether PtrA is required for PA23-mediated control of Sclerotinia stem rot. We also generated an *scd* mutant and determined its role in PA23 fungal suppression. Finally, a combination of complementation and transcriptional analysis was used to explore interactions between PtrA and other regulators of PA23 biocontrol.

## Materials and methods

### Bacterial strains and growth conditions

The bacterial strains and plasmids used in this study are listed in Table 1. *Escherichia coli* strains were cultured at 37°C on Lennox Luria Bertani (LB) agar (Difco Laboratories, Detroit, Michigan). *P. chlororaphis* PA23 and its derivatives were cultured at 28°C on LB agar or M9 minimal media supplemented with 1mM MgSO_4_ and 0.2% glucose. For AF assays, bacteria were grown on one-fifth potato dextrose agar (PDA; Difco). As required, media were supplemented with the following antibiotics: tetracycline (Tc; 15 μg/mL), gentamicin (Gm; 15 μg/mL), ampicillin (Amp; 100 μg/mL) for *E. coli*, and rifampicin (Rif; 25 μg/mL), Tc (15 or 100 μg/mL), Gm (20 μg/mL), piperacillin (Pip; 40 or 500 μg/mL) for *P. chlororaphis.* All antibiotics were obtained from Research Products International Corp. (Mt. Prospect, Illinois).

**Table 1:** **Bacterial strains, plasmids, and oligonucleotide sequences**.

**Strains**	**Relevant genotypes**	**Source or references**
***PSEUDOMONAS CHLORORAPHIS***		
PA23	Phz^+^Rif^R^ wild type (soy bean plant isolate)	Savchuk and Fernando, 2004
PA23-443	Phz^−^Rif^R^ *ptrA*::Tn*5*-OT182 genomic fusion	Klaponski et al., 2014
PA23*scd*	Tc^R^ marker inserted into *scd* gene	This study
***ESCHERICHIA COLI***		
DH5α	*supE44 ΔU169 (φ80lacZΔM15) hadR17 recA1 endA1 gyrA96 thi-1 relA1*	Gibco
DH5αλpir	DH5λpir lysogen of DH5α	House et al., 2004
***CHROMOBACTERIUM VIOLACEUM***		
CVO26	Autoinducer synthase (*cviI*) mutant from *C. violaceum* ATCC 31532, autoinducer biosensor	Latifi et al., 1995
**PLASMIDS**		
pUCP23	Broad-host range vector; Amp^R^, Gm^R^	West et al., 1994
pUCP22-*gacA*	1.65-kb fragment containing *gacA* and *uvrC* from *P. protegens* CHA0 in pUCP22	This study
pUCP23-*gacS*	3.1-kb fragment containing *gacS* in pUCP23	Poritsanos et al., 2006
pUCP22-*ptrA*	2.2-kb fragment containing *ptrA* in pUCP22	Klaponski et al., 2014
pUCP22-*rpoS*	1.3-kb fragment containing *rpoS* in pUCP22	Poritsanos et al., 2006
pUCP22-*psrA*	950-bp fragment containing *psrA* in pUCP22	This study
pUCP22-*rsmA*	190-bp fragment containing *rsmA* in pUCP22	This study
pUCP22-*rsmE*	600-bp fragment containing *rsmE* in pUCP22	This study
pUCP23-*rsmZ*	400-bp fragment containing *rsmZ* in pUCP23	This study
pUCP23-*phzR*	1.68-kb fragment containing *phzR* in pUCP23	Selin et al., 2012
pUCP22-*retS*	2.9-kb fragment containing *retS* in pUCP22	This study
pUCP22-*ladS*	2.8-kb fragment containing *ladS* in pUCP22	This study
pRK600	Mobilization plasmid containing *tra* genes, Chl^R^	Finan et al., 1986
pKNOCK-Tc	Suicide vector designed for insertional mutagenesis; R6K ori; RP4 oriT; Tc^R^	Alexeyev, 1999
pKNOCK-*scd*	542-bp internal *scd* fragment cloned into pKNOCK-Tc	This study
**OLIGONUCLEOTIDE SEQUENCES**		
tet FWD	5′- ACCCGTCCTGTGGATTCTCTA-3′	This study
new ptrA TL start FWD	5′- GCAAGCAAGCTTCGACGCGATACAACTGGC- 3′	This study
scd-pKNOCK FWD	5′- TATTGGATCCTTCCACGCTCTTGGCGTA-3′	This study
scd-pKNOCK REV	5′- TATTCTCGAGCCAACGGCACCATAGGTTCA-3′	This study
retS-F2	5′- GACGGATCCAGCGCCGCGCATAGTTAT-3′	This study
retS-R2	5′- ATGAAGCTTGGCGCAAACTCACAGCG-3′	This study
ladS-F-BamHI	5′- GAGTGGATCCAAACCAATAACAGG-3′	This study
ladS-R-HindIII	5′- CCAGAAGCTTAGTTAAGCACCC-3′	This study
gacS RT-PCR FWD	5′- TGGTCAGCCTGGTGTATC-3′	This study
gacS RT-PCR REV	5′- TGTCTTCGTGTTCTTCTTCG-3′	This study
rpoS RT-PCR FWD	5′- TGGCTTTCCGAATTGACC-3′	This study
rpoS RT-PCR REV	5′- CAGACGCTTGAGACCTTC-3′	This study
prnA RT-PCR FWD	5′- CTGTCGTCGTGCTTTCTG-3′	This study
prnA RT-PCR REV	5′- GATCTCGGCGTTGAATGC-3′	This study
phzI RT-PCR FWD	5′- GCGATGCCGTTGTTCTGG-3′	This study
phzI RT-PCR REV	5′- AGCCGTTCGTAGTGGACTC-3′	This study
phzR RT-PCR FWD	5′- GAATCCTTGGCTTCAGACC-3′	This study
phzR RT-PCR REV	5′- ATCAGGCGGCTAACTACG-3′	This study
psrA RT-PCR FWD	5′- CCATCTTCATGCGTCTTCTG-3′	This study
psrA RT-PCR REV	5′- ATGTAGCGGCGGAATACC-3′	This study
rsmZ RT-PCR FWD	5′- TGCGGTATGAAAGTTGTCTATTTG-3′	This study
rsmZ RT-PCR REV	5′- ATCCTTGATGGTTGTGTCTATCC-3′	This study
rsmE RT-PCR FWD	5′- GAAAGCATAAATATCGGTGAC-3′	This study
rsmE RT-PCR REV	5′- CGTTGGTAGATTTCTTCGC-3′	This study
phzA RT-PCR FWD	5′- GACTGGCAATGGCACAAC-3′	This study
phzA RT-PCR REV	5′- GCAATAACCTTCGGGATAACC-3′	This study
gacA RT-PCR FWD	5′- CTGGTGTTCAAGTCATTCC-3′	This study
gacA RT-PCR REV	5′- AAGATACGGTAACGGTAGG-3′	This study
rsmA RT-PCR FWD	5′- ATGCTGATTCTGACTCGTC-3′	This study
rsmA RT-PCR REV	5′- GCACCGCTACCTCTTTAG-3′	This study
rpoB RT-PCR FWD	5′- CGTGTTCCTGCCGCTATC-3′	This study
rpoB RT-PCR REV	5′- GCCGCAACCGAAACTACC- 3′	This study
ptrA RT-PCR F3	5′- ACCTGGAGCAATATGGCGAG-3′	This study
ptrA RT-PCR R3	5′- TGCTGGTGATAGAGCCACTC-3′	This study
retS RT-PCR F2	5′- AGCACCACGTCGAAGTAGTCGC-3′	This study
retS RT-PCR R2	5′- ACAACGACACCTGCCGCAAG-3′	This study
ladS RT-PCR F1	5′- AGAGGTAATCGAGCAGGCAGCG-3′	This study
ladS RT-PCR R1	5′- GCTCAAACTGTGCGACCAGGTG-3′	This study
ladS RT-PCR R1	5′- GCTCAAACTGTGCGACCAGGTG-3′	This study
Up1 RT-PCR F	5′- GCCACCGAAATAGGCGCAAC-3′	This study
Up1 RT-PCR R	5′- CCAACAACCGCCATGTCGAAC-3′	This study
Up2 RT-PCR F	5′- TTGCTCGAAGCGCACTTCAC-3′	This study
Up2 RT-PCR R	5′- AGATCCTCTACGTCAGCAAGCC-3′	This study
Up3 RT-PCR F	5′- ATTGTGGGTTCTTGCGGCTG-3′	This study
Up3 RT-PCR R	5′- CTCTGCGGGATCGGCTTCACCATGAGCCTG-3′	This study

### PCR

Polymerase Chain Reaction (PCR) was performed as follows. Each reaction contained 2.5 μL of both forward and reverse primers (12 μM), 1 μL of template DNA, 10 μL of 10 × Taq Buffer (without Mg added), 1.5 μL of MgSO_4_, 1 μL of Taq polymerase (Thermo Fisher Scientific, Carlsbad, USA) and nuclease-free water to a final volume of 100 μl. PCR reaction conditions included an initial denaturation at 98°C for 2 min, followed by 30 cycles of 98°C for 30 s, 55°C for 30 s, and 68°C for 1 min/kb, followed by a final extension of 68°C for 5 min.

### Nucleic acid manipulation

Cloning, purification, electrophoresis, and other manipulations of nucleic acid fragments and constructs were performed using standard techniques (Sambrook et al., 1989).

### Generation of a *scd* mutant

To generate PA23*scd*, a 542-bp internal fragment of the *scd* gene was PCR amplified from PA23 genomic DNA using primers scd-pKNOCK FWD and scd-pKNOCK REV. The amplicon was gel purified and digested with *Bam*HI and *Xho*I and cloned into the same sites of pKNOCK-Tc. The pKNOCK-*scd* plasmid was then mobilized into PA23 through triparental mating with the donor strain *E. coli* DH5αλpir containing pKNOCK-*scd* and the helper strain DH5α (pRK600). Pseudomonas Isolation Agar (PIA, Difco) supplemented with Tc (150 μg/mL) was used to screen for transconjugants. Insertion of the plasmid into *scd* was confirmed by PCR using primers tet FWD and new ptrA TL start FWD followed by sequencing of the amplicon.

### Plasmid construction

For complementation analysis, pUCP22-*retS* and pUCP22-*ladS* were generated as follows. *retS* was PCR amplified from PA23 genomic DNA using primers retS-F2 and retS-R2. The 2.9-kb amplicon was digested with *Bam*HI & *Hin*dIII before cloning into the same sites of pUCP22. The *ladS* gene was amplified using primers ladS-F-BamHI and ladS-R-*Hin*dIII. The 2.8-kb *ladS*-containing fragment was subject to digestion with *Bam*HI & *Hin*dIII and cloned into pUCP22 digested with the same enzymes. pUCP22-*retS* and pUCP22-*ladS* were verified through sequence analysis.

### Antifungal assays

Radial diffusion assays to assess fungal inhibition *in vitro* were performed according to previously described methods (Poritsanos et al., 2006). Five replicates were analyzed for each strain and assays were repeated three times.

### Greenhouse assays

Strains PA23 (pUCP22), PA23-443 (pUCP22), and PA23-443 (*ptrA*-pUCP22) were assessed for their ability to control stem rot of canola [*Brassica napus* (cv. Westar)] under greenhouse conditions. Canola plants were grown in pots (21 × 20 cm) at 24/16°C with a 16-h photoperiod. The plants were sprayed at 30% flowering with bacterial strains (2.0 × 10^8^ CFU mL^−1^) suspended in sterile distilled water with 0.02% Tween 20 and kept in a growth chamber (24/16°C, 16-h photoperiod). Twenty-four hours after bacterial inoculation, plants were sprayed with ascospores of *S. sclerotiorum* (8 × 10^4^ spores mL^−1^) suspended in water containing 0.02% Tween 20. Pathogen control plants were inoculated with ascopores only, while healthy control plants were sprayed with water (0.02% Tween 20). After pathogen inoculation, plants were incubated in a humidity chamber for 72 h, after which they were placed back in the growth chamber. Fourteen days after ascospore inoculation, symptom development on the stem and leaves was scored according to Selin et al. (2010). Ten plants were used for each treatment and the plant studies were repeated two times.

### Phenazine analysis

Production of PCA and 2-OH-PHZ was quantified according to the methods outlined by Chancey et al. (1999). Overnight cultures (5 mL) were grown in M9 minimal media (1 mM MgSO_4_; 0.2% glucose) and subjected to PHZ extraction. Spectrophotometric quantification was performed at 367 nm and 490 nm for PCA and 2-OH-PHZ, respectively (Maddula et al., 2008). PHZ analysis was performed in triplicate.

### Pyrrolnitrin analysis

Production of the antibiotic PRN was quantified according to the methods outlined by Selin et al. (2010). Briefly, 20 mL cultures of PA23 and its derivatives were grown for 5 days in M9 minimal media (1 mM MgSO_4_; 0.2% glucose) and PRN was extracted with an equal volume of ethyl acetate. Before extraction, toluene was added to each sample as an internal control. Toluene and PRN UV absorption maxima were recorded at 225 nm with a Varian 335 diode array detector. PRN peaks were detected at 4.7 min. Samples were analyzed in duplicate.

### Autoinducer analysis

The production of AHL was analyzed by spotting 10 μL of an overnight culture onto LB agar plates seeded with *C. violaceum* CV026. This strain is able to detect exogenous AHLs with carbon chain length structures ranging from C4 to C8, resulting in a purple halo surrounding the colonies. The diameter of the purple zones was measured at 24 h.

### Semi-quantitative reverse transcriptase PCR

To monitor expression of genes involved in biocontrol, semi-quantitative reverse transcriptase (RT) PCR was used. PA23 and its derivatives were grown to early stationary phase and total RNA was extracted using a RNeasy Mini Kit (QIAGEN, Valencia, USA). Residual genomic DNA was removed by treatment with TURBO RNAase-free DNAse I (Ambion, Carlsbad, USA) during the RNA isolation procedure. RNA concentrations were measured at 260 and 280 nm and only RNA samples with A_260_/A_280_ between 1.8 and 2.0 were used in subsequent steps. cDNA was generated by reverse transcription using the Maxima First Strand cDNA Synthesis Kit (ThermoScientific, Rockford, USA) and random hexamer primers in a 20 μL total reaction volume. The following conditions were employed: initial heating at 25°C for 10 min, 50°C for 15 min for reverse transcription and 85°C for 5 min for enzyme denaturation. Sequences for the genes of interest from PA23 were obtained from GenBank. The primer sequences are listed in Table 1. PCR was performed using a CFX96 Connect™ Real-Time PCR Detection System (Bio-rad, Hercules, USA) and SsoFast™ EvaGreen® Supermix (Bio-rad). The final 10-μL volume mixture in each well contained 0.4 μL of both forward and reverse primers (12 μM), 1 μL of 1:20 diluted cDNA, 5 μL of SsoFast™ EvaGreen® Supermix and 3.4 μL of nuclease-free water. PCR reaction conditions included an initial denaturation at 98°C for 2 min, followed by 39 cycles of 98°C for 5 s, 60°C for 30 s, and 60°C for 5 s. Melt-curve analysis was performed to evaluate the formation of primer dimers and other artifacts to validate results. Each reaction was performed in triplicate and experiments were repeated three times with three biological replicates. Relative gene expression was calculated using the ΔΔCt method as described by Livak and Schmittgen (2001) using *rpoB* as the reference gene and the CFX Manager™ software (Bio-rad).

### Phylogenetic analysis

Genetic sequences for *ptrA* homologs were obtained through BLAST (Altschul et al., 1990). The accession number for *ptrA* (EF054873.1) was used as a query against Pseudomonadales (taxid:72274) sequences in the non-redundant nucleotide collection (nr/nt) employing the blastn algorithm. For amino acid sequences, this query was used against the non-redundant protein database using the blastp algorithm. Forty nucleotide and 41 amino acid sequences were selected with *E*-values of <2 × 10^−100^. Both sets of sequences were aligned using Mafft servers (Katoh et al., 2005). Phylogenetic trees were constructed with the MEGA6 program package (Tamura et al., 2013). For nucleotide sequences, the Maximum likelihood method based on the Tamura-Nei model (Tamura and Nei, 1993) was applied, with genetic distances estimated through the Maximum composite likelihood approach. Bootstrap analysis with a thousand substitutions was utilized to test the trees. The nucleotide sequence for a LTTR from *Serratia marcescens* strain RSC-14 (gi: 926475601) was included as an outgroup for phylogenetic tree construction. For amino acid sequences, the Maximum likelihood method based on the Le-Gascuel 2008 model (Le and Gascuel, 1993) was employed with genetic distances estimated using the JTT model. Bootstrap analysis with a thousand substitutions was used to test the trees. The amino acid sequence for a LTTR from *S. marcescens* (gi: 759524346) was included as an outgroup for phylogenetic tree construction.

### Statistical analysis

All statistical analysis was performed using unpaired Students's *t*-test.

## Results

### PtrA is essential for PA23 biocontrol of *S. sclerotiorum* in the greenhouse

**Figure 1:**
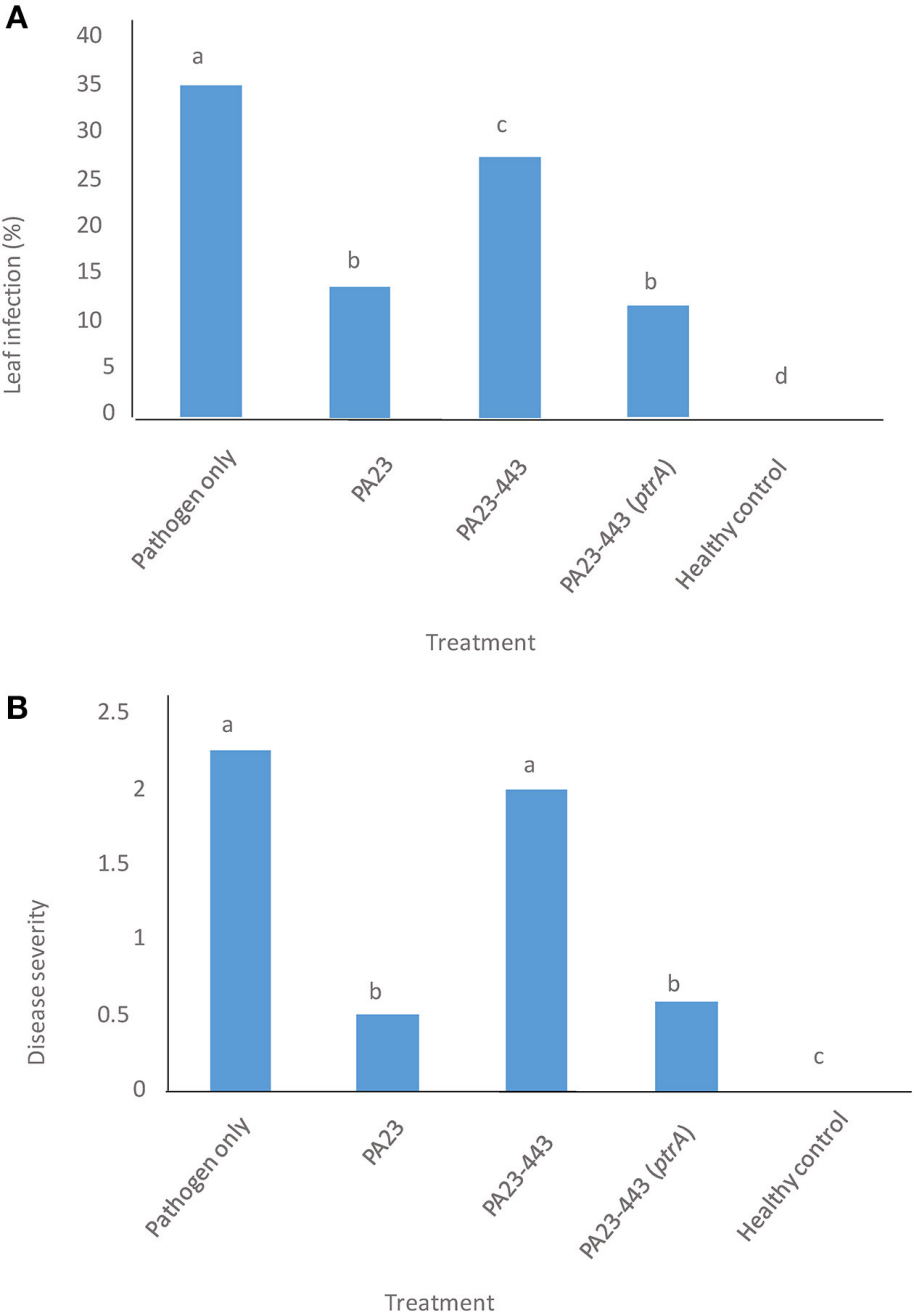
**Efficiency of *Pseudomonas chlororaphis* PA23 (wild type, pUCP22), *ptrA*-mutant PA23-443 (pUCP22), and *ptrA*-complemented strain PA23-443 (pUCP22-*ptrA*) in managing *Sclerotinia sclerotiorum* ascospore infection on canola plants. (A)** Percent incidence of leaf infection. **(B)** Stem rot disease severity. In all treatments except the healthy control, plants were sprayed with *S. sclerotiorum* ascospores (8 × 10^4^ spores/ml). The healthy control plants were sprayed with water. Column means labeled with the same letter do not differ significantly by Duncan's Multiple Range Test (DMRT; *P* > 0.05).

The wild-type PA23, PA23-443 and the complemented PA23-443 (*ptrA*-pUCP22) were tested for their ability to protect canola from stem rot disease caused by *S. sclerotiorum*. Two parameters were evaluated, namely incidence of leaf infection and stem rot disease severity. As illustrated in Figure 1, the *ptrA* mutant showed a significant reduction in its ability to control fungal infection of leaves and stems and reduce overall disease severity. Compared to the disease control, the *ptrA* mutant mediated a modest decrease in leaf infection (Figure 1A) and no difference in disease severity (Figure 1B). Addition of *ptrA in trans* restored the ability of PA23-443 to prevent both leaf infection and stem rot to wild-type levels (Figure 1).

### *scd*, which lies upstream of *PtrA*, does not appear to be involved in PA23 AF activity

**Figure 2:**
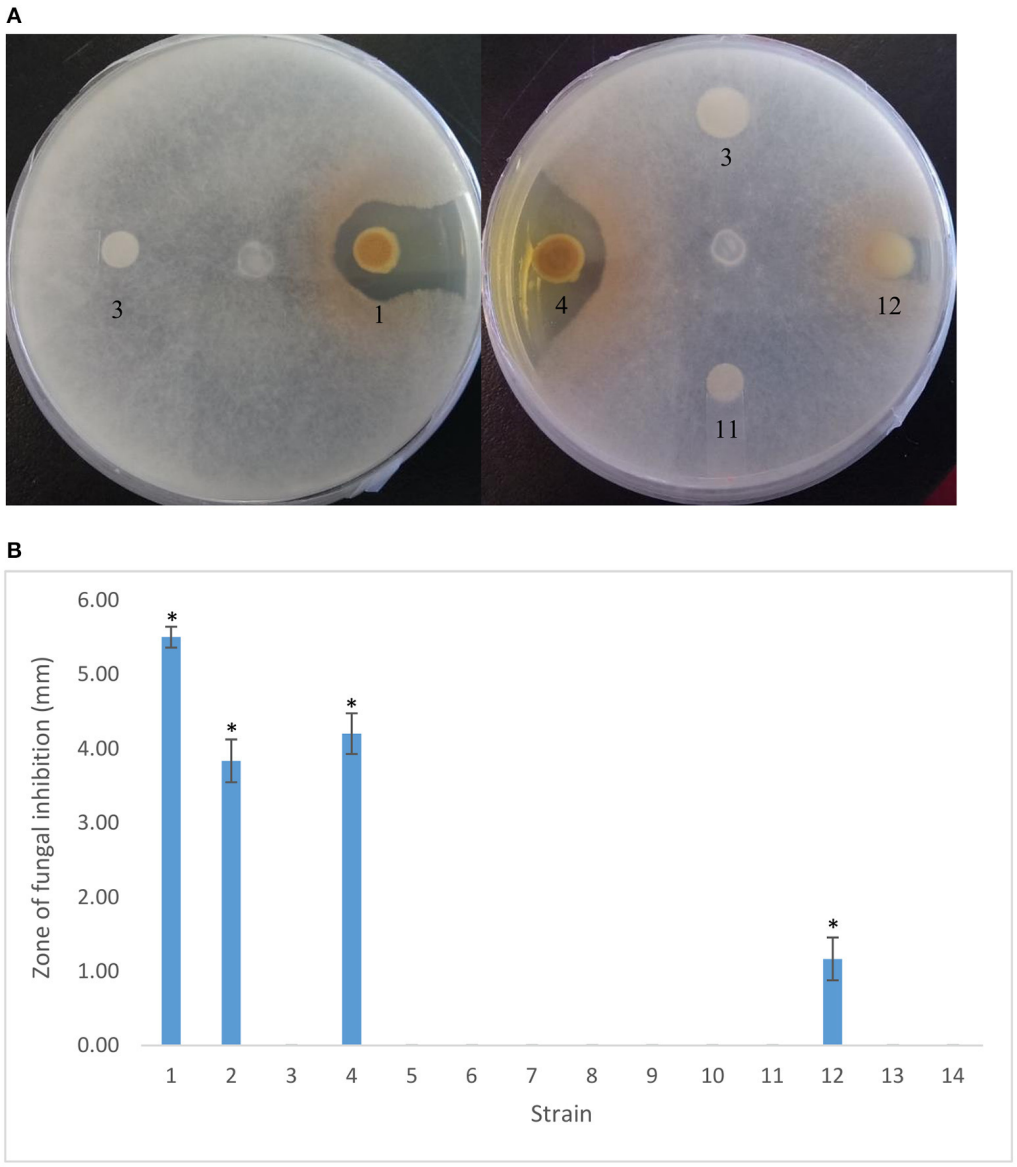
***In vitro* antifungal activity of PA23 and derivative strains against *Sclerotinia sclerotiorum*. (A)** Radial diffusion assays showing *S. sclerotiorum* inhibition after 48 h growth on 1/5 PDA at room temperature. **(B)** Zone of fungal inhibition (mm) surrounding bacterial colonies. Samples are as follows: 1, PA23 (pUCP22); 2, PA23*scd* (pUCP22); 3, PA23-443 (pUCP22); 4, PA23-443 (pUCP22-*ptrA*); 5, PA23-443 (pUCP22-*rsmA*); 6, PA23-443 (pUCP22-*rsmE*); 7, PA23-443 (pUCP23-*rsmZ*); 8, PA23-443 (pUCP22-*rpoS*); 9, PA23-443 (pUCP22-*psrA*); 10, PA23-443 (pUCP23-*phzR*); 11, PA23-443 (pUCP22-*gacA*); 12, PA23-443 (pUCP23-*gacS*); 13, PA23-443 (pUCP22-*retS*), PA23-443 (pUCP22-*ladS*). For strains that differ significantly from PA23-443 (*ptrA* mutant), columns have been marked with an asterisk (*p < 0.0001).

A 115-bp intergenic region separates *ptrA* and an upstream gene encoding a short-chain dehydrogenase, designated *scd.* To determine whether this allele is involved in PtrA regulation, an *scd* insertional mutant was generated. Unlike the *ptrA* mutant that is devoid of AF activity, the *scd* mutant showed near wild-type fungal suppression (Figure 2). Due to the plasmid insertion, only the first 100 nt (of 600) of the *scd* open reading frame remain intact (data not shown). It seems highly unlikely that a functional truncated Scd is being produced, but we cannot rule out this possibility entirely.

### *gacS* is able to partially complement the *PtrA* mutant

To reveal interactions between PtrA and other members of the regulatory hierarchy overseeing PA23 biocontrol, plasmid-borne copies of regulatory genes constitutively expressed from the *lac* promoter were transformed into the *ptrA* mutant. Genes that are able to fully or partially complement the mutant are predicted to lie downstream of PtrA in the regulatory cascade. We began our characterization of the PA23-443 transformants by analyzing AF activity. As expected, providing *ptrA in trans* restored AF activity close to wild-type levels (Figure 2). The only other gene that resulted in partial complementation of fungal suppression was *gacS* (Figure 2).

**Figure 3:**
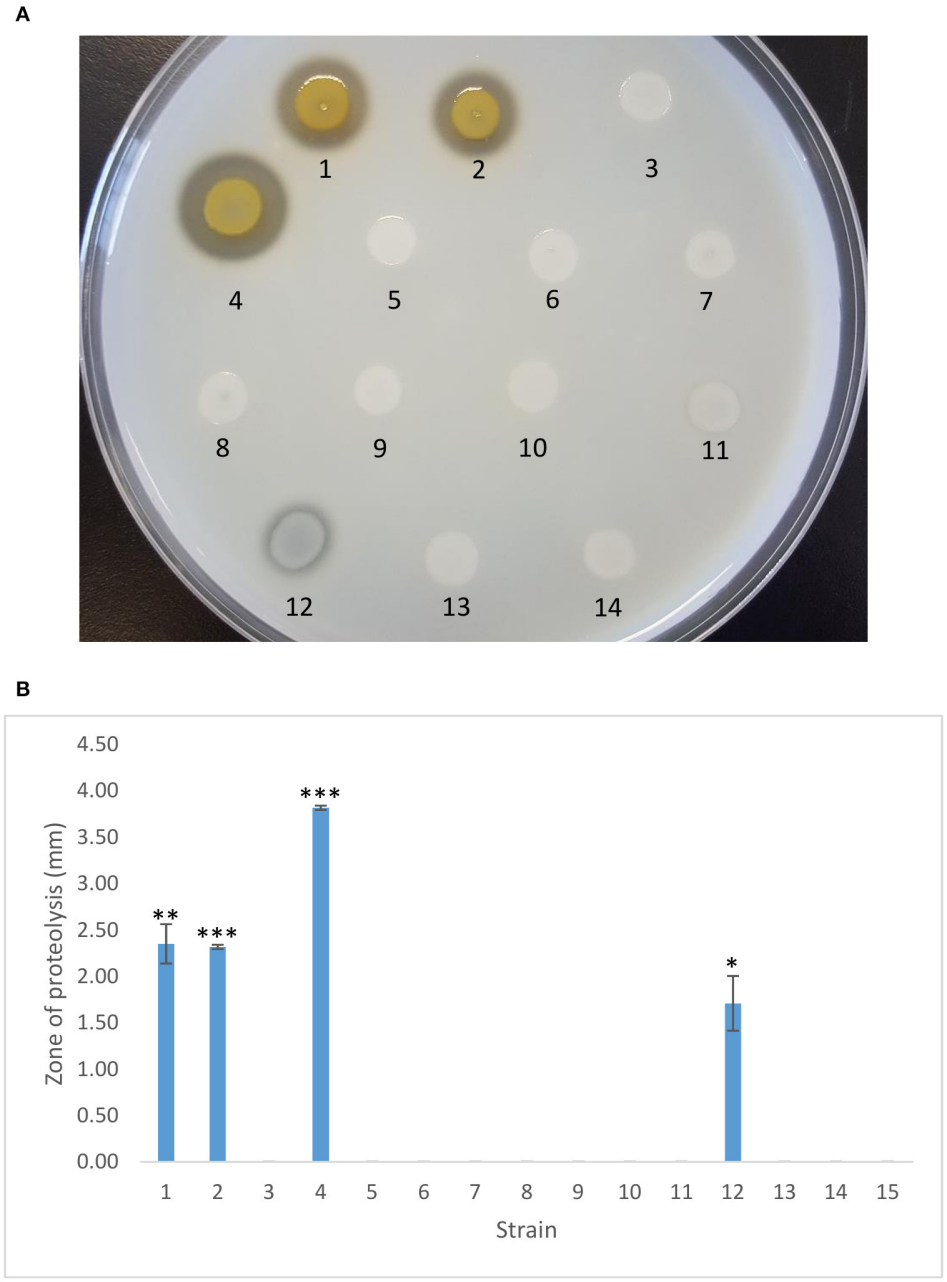
**Protease activity exhibited by PA23 and derivative strains. (A)** Protease production on 2% skim milk agar. **(B)** Zone of proteolysis (mm) surrounding bacterial colonies after 48 h incubation at 28°C. Samples are as follows: 1, PA23 (pUCP22); 2, PA23*scd* (pUCP22); 3, PA23-443 (pUCP22); 4, PA23-443 (pUCP22-*ptrA*); 5, PA23-443 (pUCP22-*rsmA*); 6, PA23-443 (pUCP22-*rsmE*); 7, PA23-443 (pUCP23-*rsmZ*); 8, PA23-443 (pUCP22-*rpoS*); 9, PA23-443 (pUCP22-*psrA*); 10, PA23-443 (pUCP23-*phzR*); 11, PA23-443 (pUCP22-*gacA*); 12, PA23-443 (pUCP23-*gacS*); 13, PA23-443 (pUCP22-*retS*), PA23-443 (pUCP22-*ladS*). For strains that differ significantly from PA23-443 (*ptrA* mutant), columns have been marked with an asterisk (*p < 0.05; **p < 0.01; ***p < 0.0001).

PA23 produces the diffusible antibiotics PHZ and PRN. Antibiotic analysis revealed that strain PA23-443 synthesized markedly lower levels of both compounds, whereas no difference in antibiotic production was observed for the *scd* mutant (Table 2). Addition of *ptrA in trans* led to partial and full restoration of PHZ and PRN production, respectively. Consistent with the AF analysis, plasmid-borne *gacS* resulted in partial complementation of antibiotic synthesis in the *ptrA* mutant (Table 2). Our protease activity profiles closely mirrored what was observed for the antibiotics. The *ptrA* mutant was devoid of protease production, whereas the *scd* mutant showed wild-type activity (Figure 3). Addition of *ptrA* and *gacS in trans* lead to full and partial rescue of protease activity in the *ptrA* mutant background, respectively (Figure 3).

**Table 2:** **Quantification of phenazines and pyrrolnitrin present in cultures of *Pseudomonas chlororaphis* PA23, PA23*scd* and PA23-443 harboring empty vector or various overexpression plasmids**.

**Strain**	**PCA^a^**	**2-OH-PHZ^a^**	**Total PHZ^a^**	**PRN^b^**
PA23 (pUCP22)	65.46 (10.3)	11.04 (2.18)	76.49 (12.47)	3.48 (0.45)
PA23*scd* (pUCP22)	77.47 (5.66)^c^	13.89 (0.60)^c^	91.35 (5.02)^c^	3.74 (0.32)^c^
PA23-443 (pUCP22)	11.04 (2.30)^d^	0.98 (0.20)^d^	12.02 (2.49)^d^	ND
PA23-443 (*ptrA*)	38.24 (4.73)^e^	5.11 (0.91)^e^	43.36 (5.60)^e^	3.90 (0.20)^f^
PA23-443 (*gacA*)	3.42 (3.25)^d^	0.45 (0.44)^d^	3.86 (5.52)^d^	ND
PA23-443 (*gacS*)	25.85 (5.91)^d^	3.92 (1.07)^d^	29.71 (6.78)^d^	2.56 (0.28)^f^
PA23-443 (*psrA*)	6.81 (1.83)^d^	0.70 (0.29)^f^	7.52 (2.10)^d^	ND
PA23-443 (*rpoS*)	4.98 (1.36)^d^	0.50 (0.21)^f^	5.48 (1.56)^d^	ND
PA23-443 (*phzR*)	10.42 (3.77)^d^	1.03 (0.48)^f^	11.46 (4.24)^d^	ND
PA23-443 (*rsmA*)	0.94 (0.82)^d^	0.02 (0.03)^d^	0.96 (0.79)^d^	ND
PA23-443 (*rsmE*)	11.42 (2.80)^d^	1.33 (0.42)^f^	12.75 (3.22)^d^	ND
PA23-443 (*rsmZ*)	11.70 (2.92)^d^	1.07 (0.34)^f^	12.77 (3.25)^d^	ND
PA23-443 (*retS*)	5.22 (1.91)^d^	0.67 (0.24)^f^	5.89 (2.15)^d^	ND
PA23-443 (*ladS*)	4.76 (0.82)^d^	0.44 (0.24)^f^	5.16 (1.06)^d^	ND

a Mean (standard deviation) of concentrations of PCA, 2-OH-PHZ and total PHZ (μg/mL) from a triplicate set.

b Mean (standard deviation) of amounts of PRN (μg) extracted from 20 ml culture volumes from a duplicate set.

c Not significantly different from wild type.

d Significantly different from wild type (P < 0.001).

e Significantly different from wild type (P < 0.05).

f Significantly different from wild type (P < 0.01).

ND, not detectable.

### PtrA regulates AHL signal production

**Figure 4:**
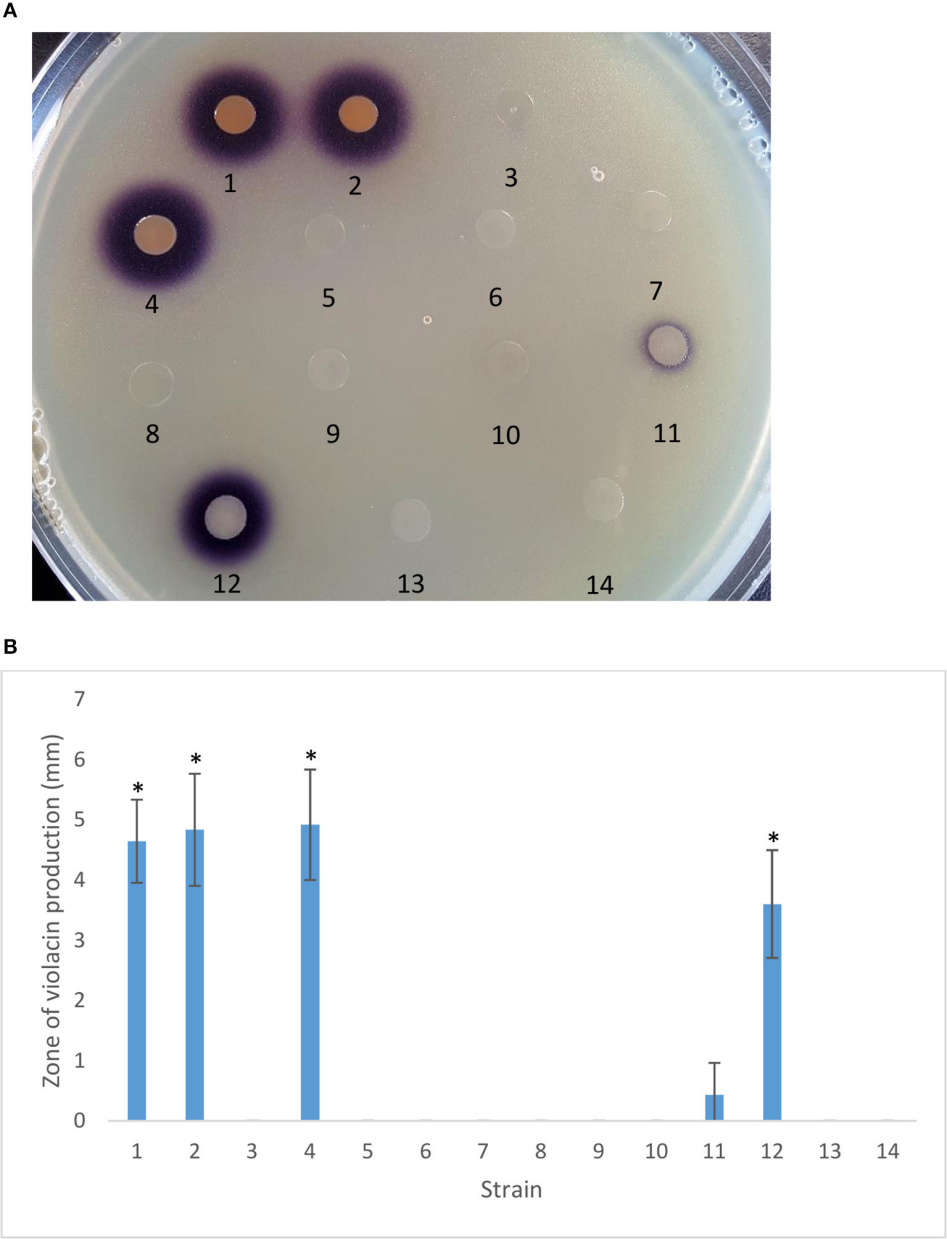
**Autoinducer production by PA23 and derivative strains. (A)** Autoinducer production by PA23, PA23-443 (*ptrA*-mutant) and derivative strains determined using *Chromobacterium violaceum* CVO26-seeded agar. **(B)** Zone of purple pigmentation (mm) indicative of bacterial autoinducer production after 48 h on CVO26-indicator plates. Samples are as follows: 1, PA23 (pUCP22); 2, PA23*scd* (pUCP22); 3, PA23-443 (pUCP22); 4, PA23-443 (pUCP22-*ptrA*); 5, PA23-443 (pUCP22-*rsmA*); 6, PA23-443 (pUCP22-*rsmE*); 7, PA23-443 (pUCP23-*rsmZ*); 8, PA23-443 (pUCP22-*rpoS*); 9, PA23-443 (pUCP22-*psrA*); 10, PA23-443 (pUCP23-*phzR*); 11, PA23-443 (pUCP22-*gacA*); 12, PA23-443 (pUCP23-*gacS*); 13, PA23-443 (pUCP22-*retS*), PA23-443 (pUCP22-*ladS*). For strains that differ significantly from PA23-443, columns have been marked with an asterisk (*p < 0.0001).

The AHL signal generated by the PhzRI QS system activates the CVO26 biosensor resulting in a white to purple color change due to the production of the QS-controlled pigment violacein. While CVO26 cells form a purple halo around colonies of PA23 and the *scd* mutant, this is not observed for PA23-443 indicating that the latter is AHL deficient. Addition of *ptrA, gacA*, and *gacS in trans* rescued AHL production in the *ptrA* mutant to varying degrees (Figure 4).

### Multiple genes show altered expression in the *PtrA* mutant

**Figure 5:**
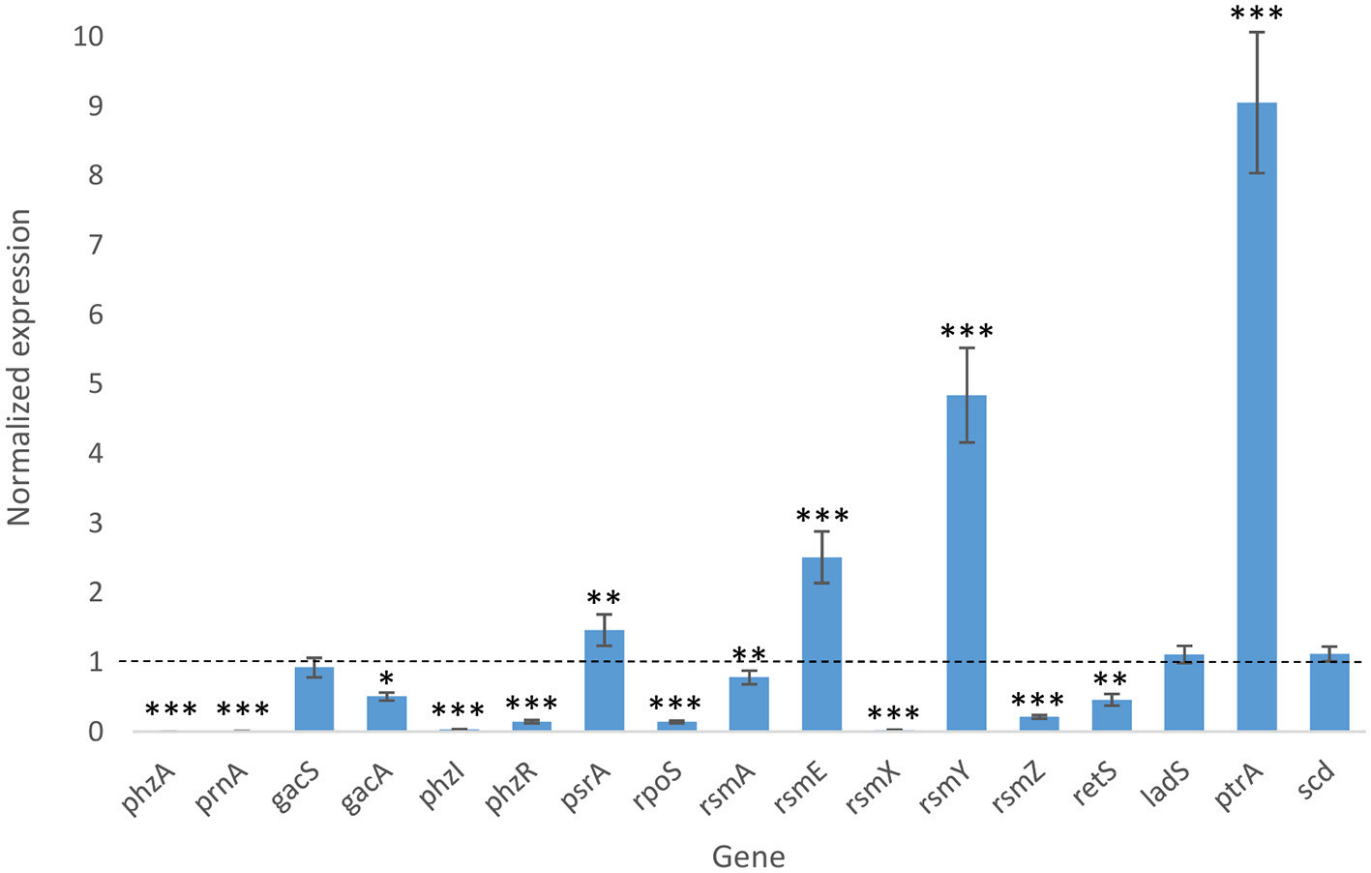
**Biocontrol gene expression in PA23-443 compared to wild type**. qRT-PCR fold change in gene expression in the *ptrA* mutant vs. wild type was determined using *rpoB* as the reference gene. The level of gene expression in the PA23 wild type was normalized to 1.0 (indicated by the dotted line). Gene expression that differs significantly from wild type is indicated with an asterisk (*p < 0.05; **p < 0.01; ***p < 0.0001).

To better understand how PtrA functions as a regulator of PA23 biocontrol, expression analysis of biosynthetic and regulatory genes associated with fungal suppression was undertaken (Figure 5). The two biosynthetic genes analyzed, *phzA* (PHZ) and *prnA* (PRN), showed a dramatic reduction in expression levels in the *ptrA* mutant background. In terms of regulatory genes, *gacA* and *gacS* exhibited a 50% reduction and no change in expression, respectively. Expression of both *phzI*, encoding the AHL synthase and *phzR*, encoding the LysR-type transcriptional regulator PhzR, were significantly downregulated in the *ptrA* mutant (Figure 5). Interestingly, *psrA* transcription was modestly increased, while the PsrA-regulated target gene *rpoS* was down regulated in the mutant background. Analysis of genes encoding Rsm repressor proteins (*rsmA, rsmE*) and regulatory RNAs (*rsmX, rsmY, rsmZ*) revealed elevated *rsmE* and *rsmY* expression in the *ptrA* mutant. Conversely *rsmA, rsmX* and *rsmZ* were downregulated, indicating positive regulation by PtrA. Next, we examined the orphan sensor kinase-encoding *retS* and *ladS* genes. A decrease in *retS* activity was observed; whereas *ladS* transcription remained at wild-type levels. The gene showing the most dramatic change in transcript abundance was *ptrA*. In the PA23-443 background, *ptrA* expression increased nine fold indicating that this LTTR is subject to negative autoregulation. No change in *scd* transcription was observed further supporting that PtrA regulation is not mediated through this divergently transcribed gene. Quantitative RT-PCR was used to monitor expression of three genes immediately upstream of *scd*, encoding a membrane protein (AIC18438.1; primers Up1), a hypothetical protein (AIC18437.1; primers Up2) as well as *nhaA* (AIC18435.1; primers Up3). No differences in transcript abundance were detected in the *ptrA* mutant compared to the wild type (data not shown).

### Phylogenetic analysis of *PtrA* homologs

**Figure 6:**
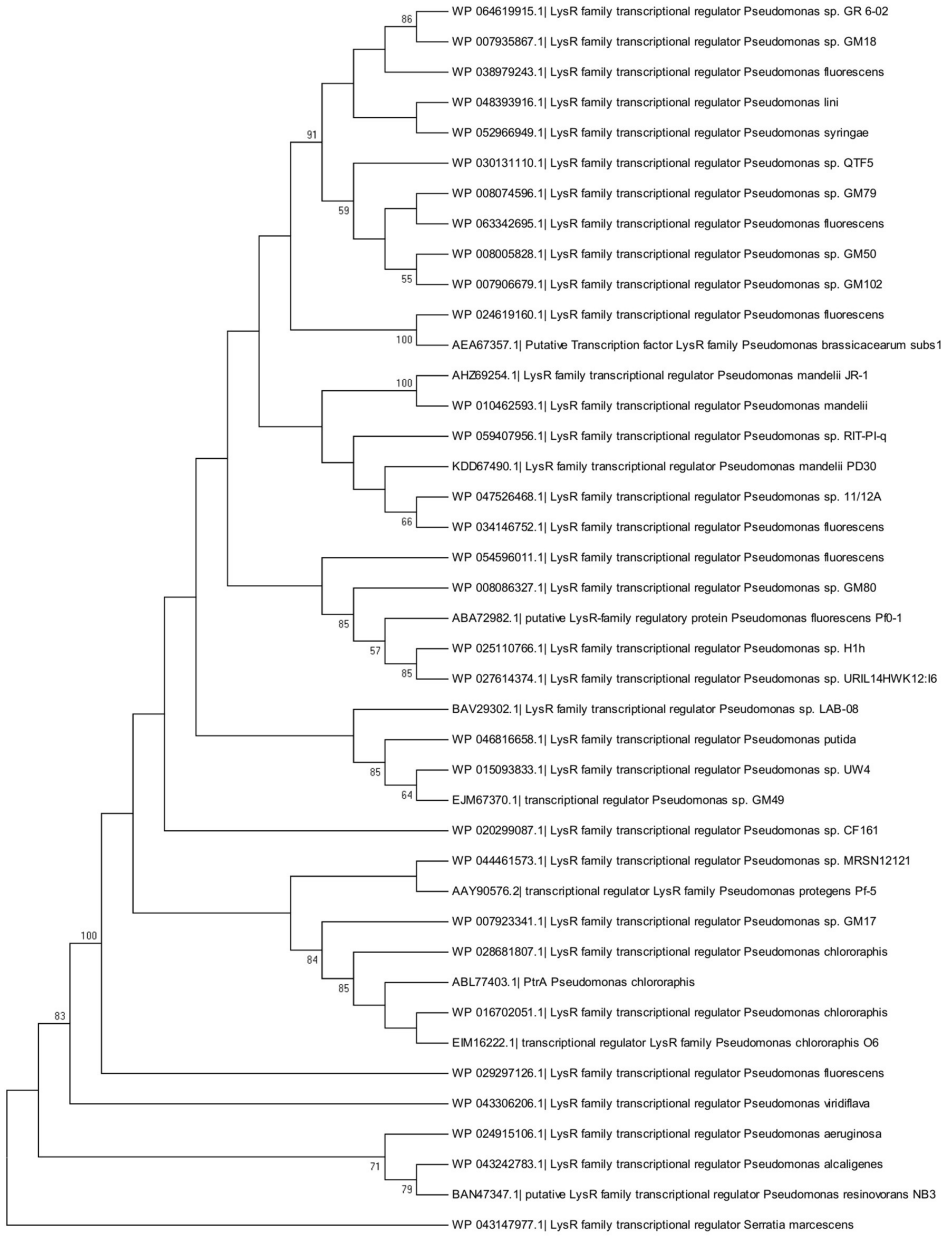
**Molecular phylogenetic analysis of PtrA homologs by the Maximum Likelihood method**. The evolutionary history was inferred using the Maximum Likelihood method based on the Le Gascuel Method (Le and Gascuel, 1993). This analysis involved 41 amino acid sequences including PtrA. The tree with the highest log likelihood is shown. The percentage of trees in which the associated taxa clustered together is indicated next to the branches.

Phylogenetic analysis revealed that PtrA was conserved amongst pseudomonads, including both biocontrol and pathogenic strains. Homologs from well-known biocontrol strains *P. chlororaphis* O6 and *P. protegens* Pf-5 were found to cluster with PtrA (Figure 6). A similar pattern was observed when a phylogenetic tree was constructed using genomic sequences (Supplementary Figure 1). As closely related homologs of PtrA are found in several biocontrol strains, they are expected to play a similar role in regulating genes responsible for the production of antifungal compounds. A list of the PtrA homologs used for the phylogenetic analysis can be found in Supplementary Tables 1, 2.

## Discussion

LTTRs, which represent the largest family of prokaryotic transcriptional regulators, frequently regulate divergently transcribed genes; however, targets can be located elsewhere on the chromosome (Schell, 1993; Maddocks and Oyston, 2008). Several pieces of evidence suggest that PtrA fits into this second paradigm, functioning as a global transcriptional regulator. First, 59 differentially regulated proteins distributed across 16 different COG categories were identified in the *ptrA* mutant (Klaponski et al., 2014) and genes encoding these proteins are scattered about the chromosome (Loewen et al., 2014). Second, insertional inactivation of the divergently transcribed *scd* gene upstream of *ptrA* resulted in no observable phenotype. AF activity, antibiotic and AHL production, and protease activity were similar to wild type (Figures 2–4; Table 2). Moreover, expression of *scd* was unchanged in the *ptrA* mutant (Figure 5). These findings indicate that *scd* is not involved in PA23 biocontrol and does not appear to be linked to PtrA. Finally, our qRT-PCR results showed altered expression of several biosynthetic and regulatory genes involved in PA23 biocontrol (Figure 5). For the most part, gene expression profiles corresponded well with the *ptrA* mutant phenotype. For example, *phzA* and *prnA* were both significantly downregulated (Figure 5). Negligible expression of both genes is consistent with the loss of orange pigmentation, fungal suppression (Figure 2) and antibiotic production (Table 2) exhibited by the *ptrA* mutant. In strain PA23, the Phz QS system regulates expression of antibiotics and degradative enzymes and so it is necessary for biocontrol (Selin et al., 2012). Transcriptional profiling revealed that *phzI* and *phzR* were significantly decreased in the *ptrA* mutant compared to the wild type. Reduced *phzI* expression coincides with the loss of AHL signal production in this background (Figure 4). While *rpoS* expression was markedly down in the *ptrA*-deficient strain, *psrA* transcription was elevated, albeit modestly (Figure 5). Because RpoS is a negative regulator of PA23 biocontrol (Manuel et al., 2012) the decrease in *rpoS* transcription was unexpected as the *ptrA* mutant is no longer capable of fungal suppression. It is important to note, however, that cross regulation occurs between the PhzRI QS system and RpoS (Selin et al., 2012), which may obscure interpretation of findings. We also explored genes belonging to the Gac-Rsm regulatory network. In *P. protegens* strain CHA0, this regulatory network functions as follows. Upon binding to an unknown signal, the sensor kinase GacS undergoes autophosphorylation and phosphotransfer to the response regulator GacA (Lapouge et al., 2008). Phosphorylated GacA can then activate expression of sRNAs, including RsmXYZ, that titrate out the translational repressors RsmA and RsmE allowing expression of target genes (Lapouge et al., 2008). While all of these components have been identified in strain PA23, the way in which they function has not yet been explored. In the closely related *P. chlororaphis* 30–84, RsmE was found to repress production of PHZ and AHL signaling molecules; however, RsmA exerted no regulatory effect over these compounds (Wang et al., 2013). Moreover, of the three small RNA molecules, only constitutively expressed *rsmZ* was able to rescue a *gacA* mutant for PHZ and AHL production; *rsmX* exhibited no effect and elevated levels of *rsmY* were reportedly lethal (Wang et al., 2013). Collectively, these findings suggest that in terms of PHZ and AHL production in *P. chlororaphis* 30–84, RsmE is the primary repressor protein and RsmZ is the small RNA responsible for lifting repression. If we assume that this circuitry functions in a similar manner in strain PA23, the elevated *rsmE* expression and reduced *rsmZ* transcription are consistent with the loss of AF activity exhibited by the *ptrA* mutant.

Even though *gacS* is not under PtrA transcriptional control, a regulatory link clearly exists between the two. Our phenotypic assays showed either full or partial complementation of the *ptrA* mutant by *gacS* when provided *in trans*. We hypothesized that PtrA might be controlling expression of regulatory elements that impact signal transduction through the Gac system and that overexpression of *gacS* is able to overcome this effect. In *P. aeruginosa* PAO1, the orphan sensor kinases RetS and LadS modulate the Gac-Rsm circuitry (Ventre et al., 2006; Goodman et al., 2009). RetS blocks GacS autophosphorylation and subsequent activation of GacA, while LadS exerts a positive effect on Gac regulation (Ventre et al., 2006; Goodman et al., 2009). To explore whether RetS and LadS are in some way linked to PtrA, we attempted to complement the *ptrA* mutant by providing *retS* and *ladS in trans*. No changes in fungal suppression, protease activity, antibiotic and AHL production were observed in the *ptrA* mutant harboring plasmid-borne copies of these genes (Figures 2–4; Table 2). We did, however, discover a two-fold decrease in *retS* transcription in PA23-443 (Figure 5). Because RetS functions to antagonize the Gac system, decreased *retS* expression is expected to have a positive impact on biocontrol. Taken together, these findings do not support a role for RetS and LadS in the PtrA -GacS interaction.

Phylogenetic analysis illustrates that a wide range of *Pseudomonas* species harbor a PtrA homolog (Figure 6). For both symbiotic and pathogenic pseudomonads, secreted products play a significant role in the biocontrol and virulence properties of these organisms. In addition, regulatory factors overseeing their expression are in many cases conserved. For example, the Gac two-component system and QS positively regulate exoproducts that play a key role in symbiotic and pathogenic interactions (Heeb and Haas, 2001; Bassler, 2002). It is not surprising, therefore, to find that PtrA is highly conserved amongst *Pseudomonas* species. To the best of our knowledge, this transcriptional regulator has not been characterized in other pseudomonads.

In summary, we have shown that PtrA is essential for biocontrol of *S. sclerotiorum* stem rot of canola. PtrA appears to function as a global regulator controlling expression of unlinked genes across the chromosome. Future studies will be directed at analyzing the PtrA transcriptome on a global scale so we can better comprehend how this LTTR is controlling expression of biocontrol factors in PA23. Due to the conserved nature of this regulator, we hypothesize that PtrA governs expression of secondary metabolites in biocontrol strains and pathogenic pseudomonads alike. Elucidating the mechanisms underlying PtrA regulation will have far-reaching implications for our understanding of how these bacteria interact with other members of their environment including prokaryotic and eukaryotic organisms.

## Author contributions

NS, NK, WF, MB, and Td conceived and designed the study. NS and NK drafted the manuscript with input from Td. NS, NK, and RR performed the phenotypic characterization of the *ptrA* mutant; CS was responsible for the greenhouse analysis. All authors read and approved the final manuscript.

### Conflict of interest statement

The authors declare that the research was conducted in the absence of any commercial or financial relationships that could be construed as a potential conflict of interest.

## Supplementary material

Supplementary Figure 1 **Molecular phylogenetic analysis of *Pseudomonas chlororaphis* PA23 *ptrA* by the Maximum Likelihood method**. The evolutionary history was inferred using the Maximum Likelihood method based on the Tamura Nei model (Tamura and Nei, 1993). This analysis involved 40 nucleotide sequences including *ptrA*. The tree with the highest log likelihood is shown. The percentage of trees in which the associated taxa clustered together is indicated next to the branches.

Supplementary Table 1 **Protein sequences used in phylogenetic analysis of PtrA and homologs**.

Supplementary Table 2 **Nucleotide sequences used in the construction of the phylogeny tree in Supplementary Figure 1**.
