# Supplementary figures and images for: PtrA Is Functionally Intertwined with GacS in Regulating the Biocontrol Activity of Pseudomonas chlororaphis PA23
Source: Front Microbiol. 2016 Sep 22;7:1512. doi: 10.3389/fmicb.2016.01512 (PMC5031690; doi:10.3389/fmicb.2016.01512)

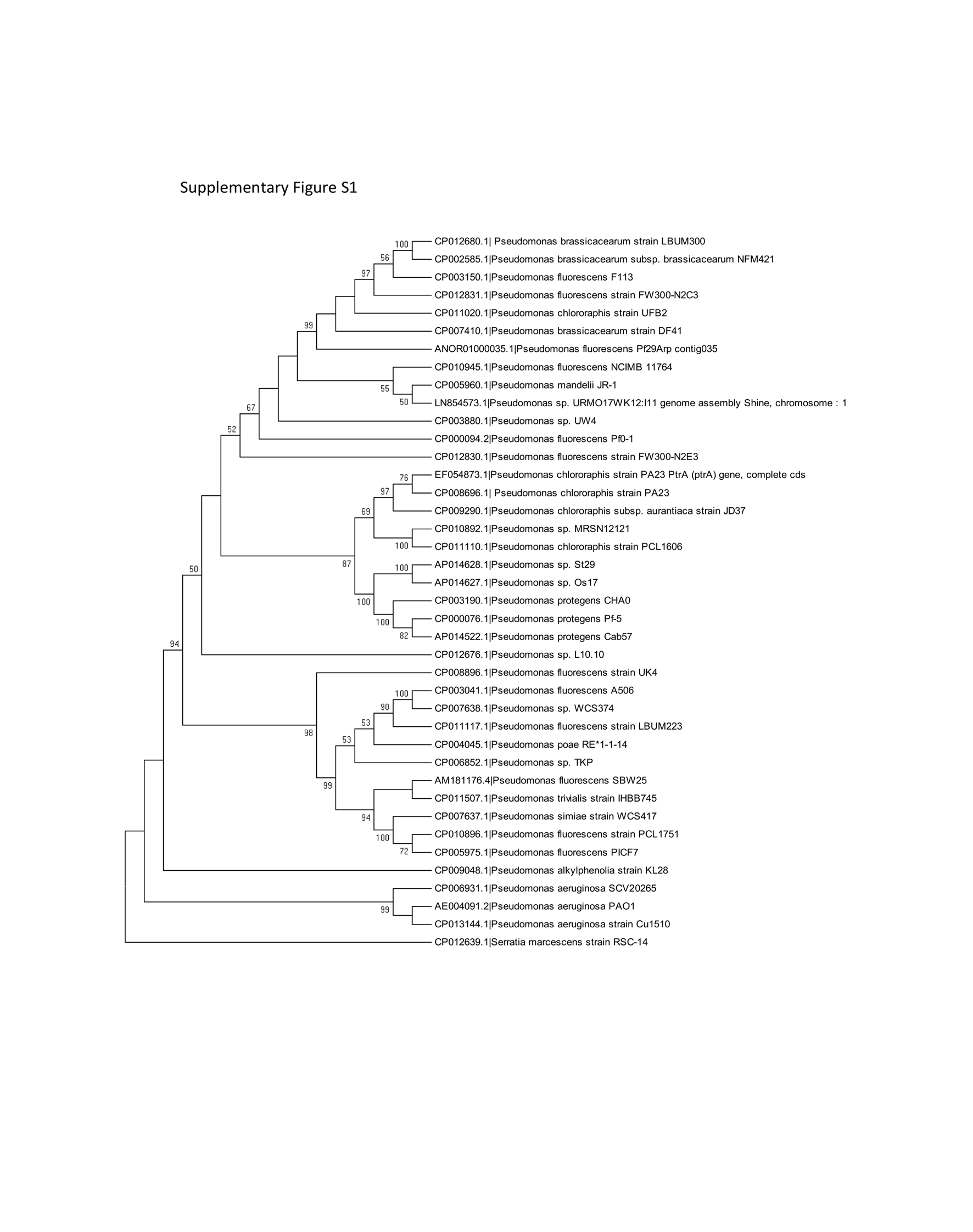

Supplement: Supplementary Figure 1 — Molecular phylogenetic analysis of Pseudomonas chlororaphis PA23 ptrA by the Maximum Likelihood method. The evolutionary history was inferred using the Maximum Likelihood method based on the Tamura Nei model (Tamura and Nei, 1993). This analysis involved 40 nucleotide sequences including ptrA. The tree with the highest log likelihood is shown. The percentage of trees in which the associated taxa clustered together is indicated next to the branches. [file Image1.TIF]
