# Supplementary material for: PtrA Is Functionally Intertwined with GacS in Regulating the Biocontrol Activity of Pseudomonas chlororaphis PA23
Source: Front Microbiol. 2016 Sep 22;7:1512. doi: 10.3389/fmicb.2016.01512 (PMC5031690; doi:10.3389/fmicb.2016.01512)
Supplement: Supplementary Table 1 — Protein sequences used in phylogenetic analysis of PtrA and homologs. [file Table1.DOCX]

**Supplemental Table 1**: Protein sequences used in phylogenetic analysis of PtrA and homologs.

| ABL77403.1 | PtrA [Pseudomonas chlororaphis] | MDDLAAFAVLIEAGSFTLAAQQLGCSKGQLSKRISALEAQFSVVLLQRTTRRLSLTAAGAALLPQAQALLVQVERARQALARLKDDMAGPVRLTVPVSLGETFFEGLLLEFSRQYPEVQIELELNNNYRDLTRDGFDLAIRSEVANDQRLVARPLLAWHEMTCASPAYLEQYGEPQTPRDLAEHRCLLNSHYSGREEWLYHQQHELLRVRVSGPFASNHYNLLKKAALVGAGIARLPSYLLQAELADGRLRGLLRDYQTRSMPMYLVHPYQYQGGLPRRTQVLADYLIGWFKRSGEALDRL |
| --- | --- | --- |
| WP_028681807.1 | LysR family transcriptional regulator [Pseudomonas chlororaphis] | MSEMDDLAAFAVLIEAGSFTLAAQQLGCSKGQLSKRISALEAQFSVVLLQRTTRRLSLTAAGAALLPQAQALLVQVERARQALARLKDDMAGPVRLTVPVSLGETFFEGLLLEFSRQYPEVQIELELNNNYRDLTRDGFDLAIRSEVANDQRLVARPLLAWHEMTCASPAYLEQYGEPQTPRDLAEHRCLLNSHYSGREEWLYHQQHELLRVRVSGPFASNHYNLLKKAALVGAGIARLPSY0LLQAELADGRLRGLLRDYQTRSMPMYLVHPYQGGLPRRTQVLADYLIGWFKRSGEALDRL |
| WP_016702051.1 | LysR family transcriptional regulator [Pseudomonas chlororaphis] | MSEMDDLAAFAVLIETGSFTLAAQQLGCSKGQLSKRISALEAQFSVVLLQRTTRRLSLTAAGAALLPQAQALLVQVERARQALARLKDDMAGPVRLTVPVSLGETFFEGLLLEFSRQYPEVQIELELNNNYRDLTRDGFDLAIRSEVANDQRLVARPLLAWHEMTCASPAYLEQYGEPQTPRDLAEHRCLLNSHYSGREEWLYHQQHELLRVRVSGPFASNHYNLLKKAALVGAGIARLPSYLLQAELADGRLRGLLRDYQTRSMPMYLVHPYQGGLPRRTQVLADYLIGWFKRSGEALDRL |
| EIM16222.1 | transcriptional regulator, LysR family [Pseudomonas chlororaphis O6] | MSEMDDLAAFAVLIEAGSFTLAAQQLGCSKGQLSKRISALEAQFSVVLLQRTTRRLSLTAAGAALLPQAQALLVQVERARQALARLKDDMAGPVRLTVPVSLGETFFEGLLLEFSRQYPEVQIELELNNNYRDLTRDGFDLAIRSEVANDQRLVARPLLAWHEMTCASPAYLEQYGEPQTPRDLAEHRCLLNSHYSGREEWLYHQQHELLRVRVSGPFASNHYNLLKKAAQVGAGIARLPSYLLQAELADGRLRGLLRDYQTRSMPMYLVHPYQGGLPRRTQVLADYLIGWFKRSGEALDRL |
| WP_007923341.1 | LysR family transcriptional regulator [Pseudomonas sp. GM17] | MSEMDDLAAFAVLIEAGSFTLAAQQLGCSKGQLSKRISALETQFAVVLLQRTTRRLSLTAAGAALLPQAQALLVQVERARQALARLKDDMAGPVRLTVPVSLGETFFEGLLLEFSRQYPEVQIELELNNNYRDLSRDGFDLAIRSEVANDQRLVARPLLAWHEMTCASPVYLEQYGEPQAPRDLAEHRCLLNSHYSGREEWLYHQQHELFRVRVSGPFASNHYNLLKKAALVGAGIARLPSYLLQAELADGRLRGLLRDYQTRSMPMYLVHPYQGGLPRRTQVLADYLIGWFKRSGEALDRL |
| WP_038979243.1 | LysR family transcriptional regulator [Pseudomonas fluorescens] | MSEMDDLAAFAVLIEAGSFTLAAQQLGCSKGQLSKRISLLETRFSVVLLQRTTRRLSLTAAGAALLPQAQALVVQVERARQALARLKDDMAGPVRMTVPVSLGETFFDGALLEFSRQYPEVQIELELNNNYRDLSRDGFDLAIRSEVANDERLVARPLLAWHEMTCASPAYLERFGEPLTPQALAEHRCLLNSHYSGREEWLYHQQHELLRVRVSGPFASNHYNLLKKAALTGAGIARLPSYLLQEELADGRLRWLLRDYQTRSMPMYLVHPYQGGLPKRTQVLADYLIGWFKRSGEALDRLRR |
| WP_048393916.1 | LysR family transcriptional regulator [Pseudomonas lini] | MSEMDDLAAFAVLIEAGSFTLAAQQLGCSKGQLSKRISLLETRFSVVLLQRTTRRLSLTAAGAALLPQAQALVVQVERARQALARLKDDMAGPVRMTVPVSLGETFFDGALLEFSRQYPEVQIELELNNNYRDLSRDGFDLAIRSEVANDERLVARPLLAWHEMTCASPAYLERFGEPLTPQALAEHRCLLNSHYSGREEWLYHQQHELLRVRVSGPFASNHYNLLKKAALTGAGIARLPSYLLQEELADGRLRWLLRDYQTRSMPMYLVHPYQGGLPKRTQVLADYLMGWFKRSGEALDRLRR |
| WP_047526468.1 | LysR family transcriptional regulator [Pseudomonas sp. 11/12A] | MSEMDDLAAFAVLVEAGSFTLAAQQLGCSKGQLSKRISQLEAQFSVVLLQRTTRRLSLTAAGAALLPQAQALVVQVERARQALARLKDDMAGPVRMTVPVSLGETFFDGLLLEFSSQYPEVQIELDLSNNYRDLSRDGYDLAVRSEVANDQRLVARPLLAWHEMTCASPAYLEQYGEPLTPQALADHRCLLNSHYSGREEWLYHQQHELLRVRVSGPFASNHYNLLKKAALAGAGIARLPSYLLPAELADGRLRWLLRDYQTRSMPMYLVHPYQGGLPKRTQVLSDYLIGWFKRSGEALDRL |
| WP_015093833.1 | LysR family transcriptional regulator [Pseudomonas sp. UW4] | MSEMDDLAAFAVLIEAGSFTLAAQQLGCSKGQLSKRISQLEAQFSVVLLQRTTRRLSLTAAGAALLPQAQALVVQVERARQALARLKDDMAGPVRMTVPVSLGETFFDGLLLEFSTQYPQVQIELELNNNYRDLSRDGFDLAIRSEVALDQRLVARPLLAWQELTCASPAYLKQYGEPQTPQALAEHRCLLNSHYSGREEWLYHQQHELLRVRVSGPFASNHYNLLKKAALVGAGIARLPSYCLPAELADGRLRWLLRDYQTRSMPMYLVHPYQGGLPKRTQVLADYLIGWFKRSGEALDRL |
| WP_030131110.1 | LysR family transcriptional regulator [Pseudomonas sp. QTF5] | MSEMDDLAAFAVLIEAGSFTLAAQQLGCSKGQLSKRISLLETRFSVVLLQRTTRRLSLTAAGAALLPQAQALVIQVERARQALARLKDDMAGPVRMTVPVSLGETFFDGALLEFSRQYPEVQIELELNNNYRDLSRDGFDLAIRSEVANDERLVARPLLAWHEMTCASPAYLERFGEPLTPQALAEHRCLLNSHYSGREEWLYHQQHELLRVRVSGPFASNHYNLLKKAALASAGIARLPSYLLQEELADGRLRWLLRDYQTRSMPMYLVHPYQGGLPKRTQVLADYLIGWFKRSGEALDRLQR |
| WP_059407956.1 | LysR family transcriptional regulator [Pseudomonas sp. RIT-PI-q] | MSEMDDLAAFAVLIEAGSFTLAAQQLGCSKGQLSKRISLLETRFSVVLLQRTTRRLSLTAAGAALLPQAQALVVQVERARQALARLKDDMAGPVRMTVPVSLGETFFDGLLLEFSGKYPEVQIELDLSNSYRDLSRDGFDLAVRSEVANDQRLVARPLLAWHEMTCASPAYLEQYSEPQTPQALAEHRCLLNSHYSGREEWLYHQQHELLRVRVSGPFASNHYSLLKKAALAGAGIARLPSYLLQAELADGRLRWLLRDYQTRSMPMYLVHPYQGGLPKRTQVLADYLIGWFKRSGEALDRLQTITRPL |
| WP_020299087.1 | LysR family transcriptional regulator [Pseudomonas sp. CF161] | MSEMDDLAAFAVLIEAGSFTLAAQQLGCSKGQLSKRISQLEAKFAVVLLQRTTRKLSLTAAGAALLPQAQALVVQVERARQALARLKDDMAGPVRITVPVSLGETFFDGLLLEFSRRYPQVQIELELNNLYRDLSRDGFDLAIRSEVANDQRLVARPLLAWRELTCASPAYLEEYGEPLAPRDLAEHRCLLNSHYSGREEWLYHQQHELLRVRVSGPFASNHYNLLKKAALAGAGVARLPSYLLQAELADGRLRWLLRDYQTRSMPMYLVHPYQGGLPKRTQVLADYLIGWFKRSGEALDRL |
| WP_008074596.1 | \| LysR family transcriptional regulator [Pseudomonas sp. GM79] | MSEMDDLAAFAVLIEAGSFTLAAQQLGCSKGQLSKRISLLETRFSVVLLQRTTRRLSLTAAGAALLPQAQALVIQVERARQALARLKDDMAGPVRMTVPVSLGETFFDGALLEFSRQYPEVQIELELNNNYRDLSRDGFDLAIRSEVANDERLVARPLLAWHEMTCASPAYLERFGEPLTPQALAEHRCLLNSHYSGREEWLYHQQHELLRVRVSGPFASNHYNLLKKAALADAGIARLPSYLLQEELADGRLRWLLRDYQTRSMPMYLVHPYQGGLPKRTQVLADYLIGWFKRSGEALDRL |
| WP_025110766.1 | LysR family transcriptional regulator [Pseudomonas sp. H1h] | MSEMDDLAAFAVLIEAGSFTLAAQQLGCSKGQLSKRISQLEAQFSVVLLQRTTRRLSLTAAGAALLPQAQALVVQVERARQALARLKDDMAGPVRMTVPVSLGETFFDGLLLEFSQKYPEVQIELELNNNYRDLSRDGFDLAIRTEVANDERLVAKPLLAWQEMTCASPAYLEQFGEPLTPQDLAEHRCLLNSHYSGREEWLYHQQHELLRVRVSGPFASNHYNLLKKAALAGAGIARLPSYLLQTELADGRLGWLLRDFQTRRMPMYLVHPYQGGLPKRTQVLADYLIGWFKRSGEALDRLQR |
| WP_008005828.1 | LysR family transcriptional regulator [Pseudomonas sp. GM50] | MSEMDDLAAFAVLIEAGSFTLAAQQLGCSKGQLSKRISLLETRFSVVLLQRTTRRLSLTAAGAALLPQAQALVIQVERARQALARLKDDMAGPVRMTVPVSLGETFFDGVLLEFSRQYPEVQIELELNNNYRDLSRDGFDLAIRSEVANDERLVARPLLAWHEMTCASPAYLERFGEPLTPQALAEHRCLLNSHYSGREEWLYHQQHELLRVRVSGPFASNHYNLLKKAALADAGIARLPSYLLQEELADGRLRWLLRDYQTRSMPMYLVHPYQGGLPKRTQVLADYLMGWFKRSGEALDRLQR |
| WP_064619915.1 | LysR family transcriptional regulator [Pseudomonas sp. GR 6-02] | MSEMDDLAAFAVLIEAGSFTLAAQQLGCSKGQLSKRISLLETRFGVVLLQRTTRRLSLTAAGAALLPQAQALVVQVERARQALARLKDDMAGPVRMTVPVSLGETFFDGALLEFSRQYPEVQIELELNNNYRDLSRDGFDLAIRSEVANDERLVARPLLAWHEMTCASPAYLERFGEPLTPQALAEHRCLLNSHYSGREEWLYHQQHELTRVRVSGPFASNHYNLLKKAALTGAGIARLPSYLLQEELADGRLRWLLRDYQTRSMPMYLVHPYQGGLPKRTQVLADYLIGWFKRSGEALDRLQR |
| WP_063342695.1 | LysR family transcriptional regulator [Pseudomonas fluorescens] | MSEMDDLAAFAVLIEAGSFTLAAQQLGCSKGQLSKRISLLETRFSVVLLQRTTRRLSLTAAGAALLPQAQALVIQVERARQALARLKDDMAGPVRMTVPVSLGETFFDGALLEFSRQYPEVQIELELNNNYRDLSRDGFDLAIRSEVANDERLVARPLLAWHEMTCASPAYLERFGEPLTPQALAEHRCLLNSHYSGREEWLYHQQHELLRVRVSGPFASNHYNLLKKAALSDAGIARLPSYLLQEELADGRLRWLLRDYQTRSMPMYLVHPYQGGLPKRTQVLADYLIGWFKRSGEALDRL |
| WP_034146752.1 | LysR family transcriptional regulator [Pseudomonas fluorescens] | MSEMDDLAAFAVLVEAGSFTLAAQQLGCSKGQLSKRISLLEAQFSVVLLQRTTRRLSLTAAGAALLPQAQALVVQVERARQALARLKDDMAGPVRMTVPVSLGETFFDGLLLEFSSQYPEVQIELDLSNNYRDLSRDGFDLAVRSDVANDERLVARPLLAWHEMTCASPAYLEQYGEPLTPQALAEHRCLLNSHYSGREEWLYHQQHELLRVRVSGPFASNHYSLLKKAALAGAGIARLPSYLLPTELADGRLRWLLRDYQTRSMPMYLVHPYQGGLPKRTQVLADYLIGWFKRSGEALDRL |
| BAV29302.1 | LysR family transcriptional regulator [Pseudomonas sp. LAB-08] | MSEMDDLAAFAVLVEAGSFTLAAQQLGCSKGQLSKRISQLEAQFSVVLLQRTTRRLSLTAAGAALLPQAQALVVQVERARQALARLKDDMAGPVRMTVPVSLGETFFDGLLLEFSARYPEVQIELELNNNYRELSRDGFDLAIRSDVANDQRLVARPLLAWHEMTCASPAYLEQFGEPQTPQALIEHRCLLNSHYSGREEWLYHQQHELLRVRVSGPFASNHYSLLKKAALAGAGIARLPSYLLPMELADGRLRWLLRDYQTRSMPMYLVHPYQGGLPKRTQVLADYLMGWFKRSGEALDRL |
| WP_054596011.1 | LysR family transcriptional regulator [Pseudomonas fluorescens] | MSEMDDLAAFAVLIEAGSFTLAAQQLGCSKGQLSKRISQLETRFSVVLLQRTTRRLSLTAAGAALLPQAQALVIQVERARQALARLKDDMAGPIRMTVPVSLGETFFDGLLLEFSREYPDVQIELELNNSYRDLSRDGFDLAIRSEVANDERMVAKPLLAWHEMTCASPAYLEQFGEPQTPADLADHRCLLNSHYSGREEWLYHQQHELLRVRVSGPFASNHYNLLKKAALAHAGIARLPSYLLQAELADGRLRWLLRDYQTRSMPMYLVHPYQGGLPKRTQVLADYLMRWFKRSGEALDRL |
| WP_052966949.1 | LysR family transcriptional regulator [Pseudomonas syringae] | MSEMDDLAAFAVLIEAGSFTLAAQQLGCSKGQLSKRISLLETRFAVVLLQRTTRRLSLTAAGAALLPQAQELVIQVERARQALARLKDDMAGPVRMTVPVSLGETFFDGALLEFSRQYPEVQIELELNNNYRDLSRDGFDLAIRSEVANDERLVARPLLAWHEMTCASPAYLERFGEPLTPQALAEHRCLLNSHYSGREEWLYHQQHELLRVRVSGPFASNHYNLLKKAALTGAGIARLPSYLLQEELADGRLRWLLRDYQTRSMPMYLVHPYQGGLPKRTQVLADYLMGWFKRSGEALDRLQR |
| AHZ69254.1 | LysR family transcriptional regulator [Pseudomonas mandelii JR-1] | MVFKWKQSMSEMDDLAAFAVLIEAGSFTLAAQQLGCSKGQLSKRISLLETRFSVVLLQRTTRRLSLTAAGAALLPQAQALVVQVEKARQALARLKDDMAGPVRMTVPVSLGETFFDGLLLEFSRQYPEVQIELDLNNSFHDLSRDGFDLAIRSEVANDQRLVARPLLAWHEMTCASPAYLEQYGEPQTPQALADHRCLLNSHYSGREEWLYHQQHELLRVRVSGPFASNHYSLLKKAALAGAGIARLPSYLLQTELADGRLRWLLRDYQTRSMPMYLVHPYQGGLPKRTQVLADYLIDWFKRSGEALARLQQ |
| WP_007906679.1 | LysR family transcriptional regulator [Pseudomonas sp. GM102] | MSEMDDLAAFAVLIEAGSFTLAAQQLGCSKGQLSKRISLLETRFSVVLLQRTTRRLSLTAAGAALLPQAQALVIQVERARQALARLKDDMAGPVRMTVPVSLGETFFDGALLEFSRQYPEVQIELELNNNYRDLSRDGFDLAIRSEVANDERLVARPLLAWHEMTCASPAYLERFGEPLTPQALAEHRCLLNSHYSGREEWLYHQQHELLRVRVSGPFASNHYNLLKKAALADAGIARLPSYLLQEELADGRLRWLLRDYHTRSMPMYLVHPYQGGLPKRTQVLADYLMGWFKRSGEALDRLQR |
| WP_046816658.1 | LysR family transcriptional regulator [Pseudomonas putida] | MSEMDDLAAFAVLIEAGSFTLAAQQLGCSKGQLSKRISQLEAQFSVVLLQRTTRRLSLTAAGAALLPQAQALVVQVERARQALARLKDDMAGPVRMTVPVSLGETFFDGLLLEFSAQYPEVQIELELNNSYRDLSRDGFDLAIRSDVAIDQRLVARPLLAWRELTCASPAYLEQYGEPQTPQALAEHRCLLNSHYSGREEWLYHQQHELLRVRVSGPFASNHYNLLKKAALAGAGIARLPSYALPEELADGRLRWLLRDYQTRSMPMYLVHPYQGGLPKRTQVLADYLIGWFKRSGEALDRL |
| WP_010462593.1 | LysR family transcriptional regulator [Pseudomonas mandelii] | MSEMDDLAAFAVLIEAGSFTLAAQQLGCSKGQLSKRISLLETRFSVVLLQRTTRRLSLTAAGAALLPQAQALVVQVEKARQALARLKDDMAGPVRMTVPVSLGETFFDGLLLEFSRQYPEVQIELDLNNSFHDLSRDGFDLAIRSEVANDQRLVARPLLAWHEMTCASPAYLEQYGEPQTPQALADHRCLLNSHYSGREEWLYHQQHELLRVRVSGPFASNHYSLLKKAALAGAGIARLPSYLLQTELADGRLRWLLRDYQTRSMPMYLVHPYQGGLPKRTQVLADYLIDWFKRSGEALARLQQ |
| WP_008086327.1 | LysR family transcriptional regulator [Pseudomonas sp. GM80] | MSEMDDLAAFAVLIEAGSFTLAAQQLGCSKGQLSKRISQLEARFSVVLLQRTTRRLSLTAAGAALLPQAQALVVQVEKARQALARLKDDMAGPVRMTVPVSLGETFFDGLLLEFSQEYPEVQIELELNNSYRDLSRDGFDLAIRSEVANDERLVAKPLLAWQEMTCASPAYLQRFGEPTTPQALAEHRCLLNSHYSGREEWLYHQQHELLRVRVSGPFASNHYNLLKKAALTGAGIARLPSYLLQAELADGRLRLLLRDYQTRRMPMYLVHPYQGGLPKRTQVLADYLIGWFKRSGEALDRLQH |
| EJM67370.1 | transcriptional regulator [Pseudomonas sp. GM49] | MDDLAAFAVLIEAGSFTLAAQQLGCSKGQLSKRISQLEAQFSVVLLQRTTRRLSLTAAGAALLPQAQALVVQVERARQALARLKDDMAGPVRMTVPVSLGETFFDGLLLEFSAKYPQVQIELELNNSYRDLSRDGFDLAIRSDVAIDQRLVARPLLAWQELTCASPAYLEQYGEPQTPQALAEHRCLLNSHYSGREEWLYHQQHELLRVRVSGPFASNHYNLLKKAALVGAGIARLPSYCLPTELADGRLRWLLRDYQTRSMPMYLVHPYQGGLPKRTQVLADYLIGWFKRSGEALDRL |
| WP_027614374.1 | LysR family transcriptional regulator [Pseudomonas sp. URIL14HWK12:I6] | MSEMDDLAAFAVLIEAGSFTLAAQQLGCSKGQLSKRISQLEAQFSVVLLQRTTRRLSLTAAGAALLPQAQALVVQVERARQALARLKDDMAGPVRMTVPVSLGETFFDGLLLEFSQKYPEVLIELELNNHYRDLSRDGFDLAIRSEVANDERLVAKPLLAWQEMTCASPAYLEQFGEPLTPQELAEHRCLLNSHYSGREEWLYHQQHELLRVRVSGPFASNHYNLLKKAALAGAGIARLPSYLLQTELADGRLRWLLRDFQTRRMPMYLVHPYQGGLPKRTQVLADYLIGWFKRSGEALDRLQR |
| WP_044461573.1 | LysR family transcriptional regulator [Pseudomonas sp. MRSN12121] | MSEMDDLAAFAVLIEAGSFTLAAQQLGCSKGQLSKRISALEAQFAVVLLQRTTRRLSLTAAGAALLPQAQALLAQVQRARQALARLKDDMAGPVRLTMPVSLGESFFDGALLAFSQTYPEVQIELELNNSYRDLARDGFDLAIRLEVANDQRLVARPLLA  WHEMTCASPAYLEQHGEPRVPQDLAGHRCLLNSHYSGREEWLYHQQHELLRVRVSGPFASNHYNLLKKAALVGAGVARLPSYLLQAELGDGRLRALLRDYQTRSMPMYLVHPYQGGLPRRTQVLADYLIDWFKRSGEALDRL |
| KDD67490.1 | LysR family transcriptional regulator [Pseudomonas mandelii PD30] | MSEMDDLAAFAVLIEAGSFTLAAQQLGCSKGQLSKRISQLESRFSVVLLQRTTRRLSLTAAGAALLPQAQALVVQVERARQALARLKDDMAGPVRMTVPVSLGETFFDGLLLEFSGKYPEVQIELDLSNSYRDLSRDGFDLAVRSEVGNDERLVARPLLAWHEMTCASPAYLEQYGEPLTPQALAEHRCLLNSHYSGREEWLYHQQHELLRVRVSGPFASNHYSLLKKAALAGAGIARLPSYLLQAELADGRLRWILRDYQTRRMPMYLVHPYQGGLPKRTQVLADYLIGWFKRSGEALDRL |
| WP_007935867.1 | LysR family transcriptional regulator [Pseudomonas sp. GM18] | MSEMDDLAAFAVLIEAGSFTLAAQQLGCSKGQLSKRISLLETRFGVVLLQRTTRRLSLTAAGAALLPQAQALVVQVERARQALARLKDEMAGPVRMTVPVSLGETFFDGALLEFSRQYPEVQIELELDNHYRDLSRDGFDLAIRSEVANDERLVARPLLAWHEMTCASPAYLERFGEPLTPQALAEHRCLLNSHYSGREEWLYHQQHELTRVRVSGPFASNHYNLLKKAALTGAGIARLPSYLLQEELADGRLRWLLRDYQTRSMPMYLVHPYQGGLPKRTQVLADYLIGWFKRSGEALDRLQR |
| ABA72982.1 | putative LysR-family regulatory protein [Pseudomonas fluorescens Pf0-1] | MVFSADKRTNGQPLVFKWKQSMSEMDDLAAFAVLIEAGSFTLAAQQLGCSKGQLSKRISQLEARFSVVLLQRTTRRLSLTAAGAALLPQAQALVVQVERARQALARLKDDMAGPVRMTVPVSLGETFFDGLLLDFSQKYPEVQIELELNNSYRDLSRDSFDLAIRTEVANDERLVAKPLLAWQEMTCASPEYLERFGEPLTPQALAEHRCLLNSHYSGREEWLYHQQHELLRVRVSGPFASNHYNLLKKAALAGAGIARLPSYLLQAELADGRLRWLLRDFQTRRMPMYLVHPYQGGLPKRTQVLADYLIGWFKLSGEALDRL |
| WP_029297126.1 | LysR family transcriptional regulator [Pseudomonas fluorescens] | MSEMDDLAAFAVLIEAGSFTLAAQQLGCSKGQLSKRISQLEAQFSVVLLHRTTRRLNLTAAGAALLPQAQALVIQVDRARQALARLKDDVAGPVRMTVPVSLGETFFYGLLLEFSRQYPQVQVEVDLSNNYRDLARDGFDLAVRSEVANDERLVAKPLLAWHEMTCASPAYLQQYGEPLTPADLVGHRCLLNSHYSGREEWLYHQQHELLRVRVDGTFASNHYNLLKKAVLVGAGIARLPSYLLQEELADGRLRWLLRDYQTRSMPMYLVHPYQGGLPRRTQVLADYLVGWFKRNGEALDRL |
| AAY90576.2 | transcriptional regulator, LysR family [Pseudomonas protegens Pf-5] | MSEMDDLAAFAVLVEAGSFTLAAQQLGCSKGQLSKRISALEARYAVVLLQRTTRRLDLTAAGAALLPQAQALVAQVERAHQALARLKDDMVGPVRLTVPVSLGETFFDGLLLEFSRHYPQVQIELDLNNSYRDLTREGFDLAVRSEVANDQRLVARPLLAWHEMTCASPAYLEQYGEPRTPQELAGHRCLLNSHYSGREEWLYHQRHELLRVRVSGPFASNHYNLLKKAALVGAGIARLPSYVLHSELADGRLRWLLRDYQTRSMPMYLVHSYQGGLPKRTQVLADYLMDWFRRSGEALDRL |
| WP_024619160.1 | LysR family transcriptional regulator [Pseudomonas fluorescens] | MSEMDDLAAFAVLMEAGSFTQAAQQLGCSKGQLSKRISLLERRFSVVLLQRTTRRLSLTAAGAALLPQAQALLVQVERARQALARLKDDISGPVRMTVPVSLGETFFEGLLMEFARTYPHVQIELELNNGYRDLTRDGFDLAIRSDAAIDERLVARPLLAWHEMTCASPAYLELYGEPETPQALAEHRCLLNSHYSGREEWLYHQQHELLRVRVSGPFASNHYSLLKKAALAGAGIARLPSYLLHEELADGRLHWLLRDYQTRRMPMYLVHPYQGGLPKRTQVLADYLIDWFKRSGEALDRLQR |
| AEA67357.1 | Putative Transcription factor, LysR family [Pseudomonas brassicacearum subsp. brassicacearum NFM421] | MSEMDDLAAFAVLMKAGSFTQAAQQLGCSKGQLSKRISLLERRFSVVLLQRTTRRLSLTAAGAALLPQAQALLVQVERARQALARLKDDISGPVRMTVPVSLGETFFEGLLMEFARTYPNVQIELELNNGYRDLTRDGFDLAIRSDAAIDERLVARPLLAWHEMTCASPAYLERYGEPETPQALAEHRCLLNSHYSGREEWLYHQQHELLRVRVSGPFASNHYSLLKKAALAGAGIARLPSYLLHEELADGRLHWLLRDYQTRRMPMYLVHPYQGGLPKRTQVLADYLIDWFKRSGEALDRLQR |
| WP_043306206.1 | LysR family transcriptional regulator [Pseudomonas viridiflava] | MNEMEDLAAFAVLIDAGSFTAAAERLGCSKGRLSKRISQLEKTYGVQLLHRTTRTLSLTSAGSALLPQARQLIAHTERARSIVALMRDAMVGEVRITTPVSLGETFFDGLLMEFYETYPQVKIELELSNSVRDLRRDGFDLAVRSKVASDERLVARPLLAMQELTCASTRYLDEHGWPQTPEDLAKHQCLINSHYSERYQWLYHQNHELTRVQIDGPFASNHYSLLKKAALAGAGVARLPSYMVHREINEGRLHWLLKDYQTATTPMFLVHPFEGEVPRRVQVLADYLVGWFERSGSALERLA |
| WP_043242783.1 | LysR family transcriptional regulator [Pseudomonas alcaligenes] | MSEMDDLAAFAVLLEVGSFTGAAERLGCSKGQLSKRMRQLEQGLGATLLHRTTRRLDLTAAGAALLPEAQALLAQANRARQAVQRLQEEIAGRVRITVPVSLGETFFDALLLEFTRRYPEVRVELDLSNSYRDLVGEGYDLGVRSGPHLDERLVARPLFSLEEITCAAPAYLARHGEPQAPADLAGHQCLLNTHYAGFEEWLYHRQHQLERVSVAGNLASNHYSLLKKAALSGAGIARLPSYMLHDELADGRLVWLLRGYQTRQSPVFLVHPFQGGLPRRTQVLMDYLLDWFERSRRALDRLGL |
| BAN47347.1 | putative LysR family transcriptional regulator [Pseudomonas resinovorans NBRC 106553] | MSEMDDLAAFAVLMEAGSFTAAAQRLGCSKGQLSKRIRLLEQGLGAALLHRTTRRLDLTAAGAALLPEAQALAAQAERARQSVLRLQEDLAGCVRLTVPVSLGETFFDALLLDFTRQYRQIRIELDLHNGYRDLVADGFDLAIRSGTDLDERLVARPLFSLQEITCATPAYLAAHGEPLKPADLATHQCLLNTHYSGFEEWLYHRQHQLERVKVAGALASNHYSLLKKAALTGAGIARLPSYLVYDELADGRLVWLLRDYQTRQTPVFLVHPWQGGLPRRTQVLVDYLLGWFERSRRMLDQVEKP |
| WP_024915106.1 | LysR family transcriptional regulator [Pseudomonas aeruginosa] | MKDLDDLAAFAVLHDLGSFTRAAERLGCSKGQLSKRIGVLEQKLGVTLLHRTTRRLSLTAAGAALLPEAQALLVQAERARQAVARLQERAEGRVRVTLPVSLGETLFDALLEDFQERHPLLRVELDLYNGMRDLVGEGFDLAIRSGVEQDARLVARPLFVLQEITCASPAYLARHGEPQRPAELAERECLLNSHYSGHEEWLYHRRHRLERVRVSGFLASNHYSLLKKAALAGTGIARLPSYMVHDELSDGRLAWLLRDYQTRSTPMFLVHPFQGGLPRRTQVLADYLLDWFERSRRKLIGLEG |
| WP_043147977.1 | LysR family transcriptional regulator [Serratia marcescens] | MLATHEYANDLILFALIVDCGSFSKAAESADITSSVISKRIGRLEKSLGARLLYRTTRSLTLTESGQALYQQAKEIGAKVQEALYAVSEKSEELTGTIRMSVPTISGELLLSESVAEFCALHPSLKVEMRLENRFVDLVEEGIDLAIRTGTMPDSSLIARPIFDSRWVIVCSPGYLESHPEPRSAEDLLGHNCLTYTYQESGTANWLMKRPGRNEIYELQVNGNLSANNARAIRKAVIGG  HGIAMVPRCMVYEDLQDGKLTEILAGHCGKVLGIYAVYPYTRNLPLKTRLLIEHIIGSYQNISHYF |
