# Supplementary material for: PtrA Is Functionally Intertwined with GacS in Regulating the Biocontrol Activity of Pseudomonas chlororaphis PA23
Source: Front Microbiol. 2016 Sep 22;7:1512. doi: 10.3389/fmicb.2016.01512 (PMC5031690; doi:10.3389/fmicb.2016.01512)
Supplement: Supplementary Table 2 — Nucleotide sequences used in the construction of the phylogeny tree in Supplementary Figure 1. [file Table2.DOCX]

**Supplementary Table 2: Nucleotide sequences used in the construction of the phylogeny tree in Figure S1.**

| **EF054873.1** | *Pseudomonas chlororaphis* strain PA23 PtrA (*ptrA*) gene | ATGGATGACCTGGCGGCGTTCGCCGTGCTGATCGAGGCCGGCAGTTTCACCCTGGCCGCCCAGCAATTGGGTTGCAGCAAGGGGCAATTGTCCAAGCGCATCAGCGCCCTGGAGGCGCAGTTCTCGGTCGTGCTGTTGCAGCGCACCACGCGCCGCTTGAGCCTGACCGCGGCGGGCGCCGCCTTGTTGCCCCAGGCCCAGGCGCTGCTGGTCCAGGTCGAGCGGGCGCGCCAGGCGCTGGCGCGGCTCAAGGACGACATGGCCGGGCCGGTGCGGCTGACGGTGCCGGTGTCCCTGGGGGAGACCTTTTTCGAGGGCCTGTTGCTGGAGTTTTCCCGGCAGTATCCCGAGGTGCAGATCGAGCTGGAGCTGAACAACAACTACCGCGACCTGACCCGGGACGGCTTCGACCTGGCGATCCGCTCGGAGGTGGCCAACGACCAGCGGCTGGTGGCCAGGCCGCTGCTGGCCTGGCACGAGATGACCTGCGCCAGCCCGGCTTACCTGGAGCAATATGGCGAGCCGCAGACGCCCAGGGACCTGGCCGAGCATCGCTGCCTGCTCAACAGTCATTACAGCGGTCGTGAGGAGTGGCTCTATCACCAGCAGCACGAGCTGTTGCGGGTGCGGGTGTCGGGGCCCTTCGCCAGCAATCACTACAACCTGCTGAAAAAGGCCGCGCTGGTGGGCGCCGGGATCGCCCGACTGCCGTCCTACCTGTTGCAGGCGGAACTGGCCGATGGGCGTTTGCGCGGGCTGCTGCGCGACTACCAGACCCGCAGCATGCCGATGTACCTGGTGCACCCGTATCAGTATCAGGGCGGGCTGCCCAGGCGCACCCAGGTCCTGGCCGATTACCTGATCGGCTGGTTCAAGCGCAGCGGCGAAGCGCTGGATCGCCTCTGA |
| --- | --- | --- |
| **CP008696.1** | *Pseudomonas chlororaphis* strain PA23 | ATGGATGACCTGGCGGCGTTCGCCGTGCTGATCGAGGCCGGCAGTTTCACCCTGGCCGCCCAGCAATTGGGTTGCAGCAAGGGGCAATTGTCCAAGCGCATCAGCGCCCTGGAGGCGCAGTTCTCGGTCGTGCTGTTGCAGCGCACCACGCGCCGCTTGAGCCTGACCGCGGCGGGCGCC  GCCTTGTTGCCCCAGGCCCAGGCGCTGCTGGTCCAGGTCGAGCGGGCGCGCCAGGCGCTGGCGCGGCTCAAGGACGACATGGCCGGGCCGGTGCGGCTGACGGTGCCGGTGTCCCTGGGGGAGACCTTTTTCGAGGGCCTGTTGCTGGAGTTTTCCCGGCAGTATCCCGAGGTGCAGATC  GAGCTGGAGCTGAACAACAACTACCGCGACCTGACCCGGGACGGCTTCGACCTGGCGATCCGCTCGGAGGTGGCCAACGACCAGCGGCTGGTGGCCAGGCCGCTGCTGGCCTGGCACGAGATGACCTGCGCCAGCCCGGCTTACCTGGAGCAATATGGCGAGCCGCAGACGCCCAGGGAC  CTGGCCGAGCATCGCTGCCTGCTCAACAGTCATTACAGCGGTCGTGAGGAGTGGCTCTATCACCAGCAGCACGAGCTGTTGCGGGTGCGGGTGTCGGGGCCCTTCGCCAGCAATCACTACAACCTGCTGAAAAAGGCCGCGCTGGTGGGCGCCGGGATCGCCCGACTGCCGTCCTACCTG  TTGCAGGCGGAACTGGCCGATGGGCGTTTGCGCGGGCTGCTGCGCGACTACCAGACCCGCAGCATGCCGATGTACCTGGTGCACCCGTATCAGGGCGGGCTGCCCAGGCGCACCCAGGTCCTGGCCGATTACCTGATCGGCTGGTTCAAGCGCAGCGGCGAAGCGCTGGATCGCCTCTGA |
| **CP009290.1** | *Pseudomonas chlororaphis subsp. aurantiaca* strain JD37 | TCAGAGGCGATCCAGCGCTTCGCCACTGCGCTTGAACCAGCCGATCAGGTAATCGGCCAGGACCTGGGTGCGCCTGGGCAGCCCGCCCTGATACGGGTGCACCAGGTACATCGGCATGCTGCGGGTCTGGTAGTCGCGCAGCAGCCCGCGCAAACGCCCGTCGGCCAGTTCCGCCTGCAA  CAGGTAGGACGGCAGTCGGGCGATCCCGGCGCCCACCAGCGCGGCCTTTTTCAGCAGGTTGTAGTGATTGCTGGCGAAGGGCCCCGACACCCGCACCCGCAACAGTTCGTGCTGCTGGTGATAGAGCCACTCCTCACGACCGCTGTAATGACTGTTGAGCAGGCAACGATGCTCGGCCAG  GTCCCTGGGCGCCTGCGGCTCGCCATACTGTTCCAGGTAGGCCGGGCTGGCGCAGGTCATCTCGTGCCAGGCCAGCAGCGGCCTGGCCACCAAGCGCTGGTCATTGGCCACCTCCGAGCGGATCGCCAGGTCGAAGCCGTCCCGGGTCAGGTCGCGGTAGTGGTTGTTCAGCTCCAGCTC  GATCTGCACCTCGGGATACTGCCGGGAAAACTCCAGCAACAGACCTTCGAAGAAGGTCTCCCCCAAGGACACCGGCACCGTCAGCCGCACCGGCCCGGCCATGTCGTCCTTGAGCCGCGCCAGGGCCTGGCGCGCCCGCTCGACCTGGACCAGCAGCGCCTGGGCCTGGGGCAACAAGGC  GGCGCCCGCCGCGGTCAGGCTCAAGCGCCGCGTGGTGCGCTGCAACAACACGACCGAAAACCGCGCCTCCAGGGCGCTGATGCGCTTGGACAATTGCCCCTTGCTACAACCCAACTGCTGGGCGGCCAGGGTGAAACTACCGGCCTCGATCAGCACGGCGAACGCCGCCAGGTCATCCAT |
| **CP010892.1** | *Pseudomonas* sp. MRSN12121 | ATGGATGACCTGGCAGCCTTCGCTGTCCTGATCGAAGCCGGCAGCTTCACCCTGGCCGCCCAGCAACTGGGCTGCAGCAAGGGGCAATTGTCCAAGCGCATCAGCGCGCTCGAGGCGCAATTCGCGGTGGTCTTGCTGCAACGCACCACCCGCCGCCTGAGCCTGACCGCGGCGGGCGCG  GCGCTGCTGCCCCAGGCCCAGGCGCTGCTGGCGCAGGTGCAGCGGGCGCGCCAGGCGCTGGCACGGCTCAAGGACGACATGGCCGGGCCGGTGCGGCTGACGATGCCGGTGTCCCTGGGGGAGAGCTTTTTCGATGGCGCGCTGCTGGCGTTTTCCCAGACCTACCCCGAGGTGCAGATC  GAGCTGGAATTGAACAACAGTTACCGCGACCTGGCCCGGGACGGTTTCGACCTGGCGATCCGCCTGGAGGTGGCCAACGACCAGCGGCTGGTGGCCCGGCCGCTGCTGGCCTGGCATGAGATGACCTGCGCCAGCCCGGCCTACCTGGAGCAGCATGGCGAGCCGCGCGTACCCCAGGAC  CTGGCCGGGCATCGCTGCCTGCTCAACAGCCACTACAGCGGCCGCGAGGAGTGGCTTTACCACCAGCAGCACGAATTGCTGCGGGTGCGGGTGTCCGGGCCTTTTGCCAGCAATCACTACAACCTGCTGAAGAAGGCCGCGCTGGTGGGGGCCGGGGTCGCTCGGCTGCCGTCCTACCTG  CTGCAGGCGGAACTGGGCGACGGGCGCCTGCGTGCCCTGCTGCGCGACTACCAGACCCGCAGCATGCCGATGTACCTGGTGCACCCGTACCAGGGCGGCTTGCCCAGGCGCACCCAGGTCCTGGCCGATTACCTGATCGACTGGTTCAAGCGCAGCGGCGAGGCGCTGGACCGCCT |
| **CP011110.1** | *Pseudomonas chlororaphis* strain PCL1606 | AGGCGATCCAGCGCCTCGCCGCTGCGCTTGAACCAGTCGATCAGGTAATCGGCCAGGACCTGGGTGCGCCTGGGCAAGCCGCCCTGGTAGGGGTGCACCAGGTACATCGGCATGCTGCGGGTCTGGTAGTCGCGCAACAGGGCACGCAAACGCCCGTCGGCCAGTTCCGCCTGCAGCAGG  TAGGACGGCAGCCGAGCGACCCCGGCCCCCACCAGCGCCGCCTTCTTCAGCAGGTTGTAGTGATTGCTGGCGAAAGGCCCGGACACCCGCACCCGTAGCAATTCGTGCTGCTGGTGGTAAAGCCACTCTTCGCGGCCGCTGTAGTGGCTGTTGAGCAGGCAGCGATGCCCGGCCAGGTCC  TGGGGTACGCGCGGCTCGCCATGCTGCGCCAGGTAGGCCGGGCTGGCGCAGGTCATCTCATGCCAGGCCAGCAGGGGCCGGGCCACCAGCCGCTGGTCGTTGGCCACCTCCAGGCGGATCGCCAGGTCGAAACCGTCCCGGGCCAGGTCGCGGTAACTGTTGTTCAATTCCAGCTCGATC  TGCACCTCGGGGTAGGCCTGGGAAAACGCCAGCAGCGCGCCATCGAAAAAGCTCTCCCCCAGGGACACCGGCATCGTCAGCCGCACCGGCCCGGCCATGTCGTCCTTGAGCCGTGCCAGCGCCTGGCGCGCCCGGTGTACCTGCGCCAGCAACGCCTGGGCCTGGGGCAGCAGCGCCGCG  CCCGCCGCGGTCAGGCTCAGGCGGCGGGTGGTGCGTTGCAGCAAGACCACCGCGAACTGCGCCTCGAGCGCGCTGATGCGCTTGGACAATTGCCCCTTGCTGCAGCCCAGTTGCTGGGCGGCCAGGGTGAAGCTGCCGGCTTCGATCAGGACGGCGAAGGCCGCCAGGTCATCCAT |
| **AP014627.1** | *Pseudomonas* sp. Os17 | ATGGATGACCTGGCGGCGTTTGCCGTGCTGGTGGAGGCGGGCAGCTTTACCCTGGCGGCCCAGCGCCTGGGTTGCAGCAAGGGCCAGTTGTCCAAGCGCCTCAGCGCCCTGGAGGCACGCTACGCCGTGGTGCTGTTGCAACGCACCACCCGGCGCCTGGACCTGACTGCCGCGGGCGCG  GCGTTGCTGCCCCAGGCCCAGGCCCTGGTTGCCCAAGTGGAACGAGCGCATCAGGCCCTGGCGCGGCTCAAGGACGACATGGCCGGGCCCGTGCGCCTGACCGTGCCGGTGTCCTTGGGCGAAACCTTTTTCGACGGCCTGCTGCTGGAGTTCTCCCGGCACTACCCCCAGGTGCAGATC  GAGCTGGACCTGAACAACAGCTACCGCGACCTGGCCCGGGAAGGCTTTGACCTGGCCGTGCGTTCCGAGGTGGCCAGCGACCAGCGCCTGGTGGCCCGGCCGCTGCTGGCCTGGCATGAAATGACCTGCGCCAGCCCGGCTTACCTGGAGCAATTCGGTGAGCCGCAGACTCCCGCGGAG  CTGGCCGAGCATCGCTGCCTGCTCAACAGTCACTACAGTGGGCGGGAGGAGTGGCTCTATCACCAGCGTCACGAGCTGCTGCGGGTGCGGGTCTCCGGGCCCTTTGCCAGCAATCACTACAACCTGCTGAAGAAGGCCGCCCTGGTGGGGGCGGGAATAGCCCGGCTGCCGTCCTATGTG  CTGCACAGCGAGCTGGCCGATGGCCGCTTGCGCTGGCTGTTGCGGGACTACCAGACCCGCAGCATGCCGATGTACCTGGTGCACCCCTATCAGGGCGGCCTGCCCAGGCGCACCCAGGTCCTGGCCGATTACCTGATGGACTGGTTCCGGCGCAGCGGTGAGGCGCTGGATCGTCT |
| **AP014522.1** | *Pseudomonas protegens* Cab57 | ATGGATGACCTGGCGGCATTTGCGGTGCTGGTGGAAGCCGGCAGTTTTACCTTGGCGGCCCAGCAGTTGGGGTGCAGCAAGGGCCAGCTCTCCAAACGCATCAGCGCCCTGGAAGCCCGCTACGCCGTGGTACTGCTGCAACGCACCACCCGGCGCCTGGACCTCACGGCTGCCGGCGCG  GCGTTGCTACCCCAGGCCCAGGCCCTGGTGGCTCAGGTCGAGCGGGCACACCAGGCCCTGGCGCGGCTCAAGGACGATATGGCCGGGCCGGTGCGCCTCACCGTCCCGGTCTCCCTGGGGGAAACCTTTTTCGACGGCCTGCTGCTGGAGTTCTCCCGGCACTATCCCCAGGTGCAGATC  GAGCTGGACCTGAACAACAGCTACCGCGACCTGACCCGGGAGGGTTTCGATCTGGCCGTGCGCTCGGAGGTGGCCAACGAGCAGCGCCTGGTGGCCCGGCCGCTGCTGGCCTGGCATGAAATGACCTGCGCCAGCCCAGCCTACCTGGAGCACTACGGCGAGCCGCGCACGCCTCAGGAG  CTGGCCGGGCATCGCTGCCTGCTCAATAGCCATTACAGCGGTCGGGAAGAGTGGTTGTACCACCAGCGCCATGAACTGTTGCGGGTACGGGTCAGCGGCCCCTTCGCCAGCAACCACTACAACCTGCTGAAGAAGGCTGCCCTGGTGGGCGCGGGGATCGCCCGGCTGCCGTCCTATGTG  CTGCACAGCGAACTGGCGGACGGCCGCCTGCGCTGGCTGCTGCGGGACTACCAGACCCGCAGCATGCCGATGTACCTGGTGCACTCCTATCAGGGCGGCCTGCCCAAGCGTACCCAGGTGCTGGCCGATTACCTGATGGACTGGTTTCGTCGCAGCGGTGAGGCGCTGGATCG |
| **CP000094.2** | *Pseudomonas fluorescens* Pf0-1 | ATGGATGATCTGGCGGCGTTCGCGGTGTTGATCGAAGCCGGCAGTTTTACCTTGGCGGCGCAGCAATTGGGGTGCAGCAAGGGGCAGTTGTCCAAGCGCATCAGTCAGTTGGAGGCGCGGTTTTCCGTGGTGTTGCTGCAACGCACCACGCGTCGGCTGAGCCTGACGGCGGCCGGTGCG  GCGTTGTTGCCACAGGCTCAGGCGCTTGTCGTCCAGGTGGAGCGAGCGCGTCAGGCATTGGCGCGATTGAAGGACGACATGGCCGGCCCGGTGCGTATGACGGTTCCGGTGTCGCTGGGGGAAACCTTCTTCGACGGTCTGCTGCTGGATTTCTCGCAGAAATACCCCGAGGTACAGATC  GAACTGGAGCTGAATAACAGCTACCGCGACCTGTCTCGCGACAGTTTCGATCTGGCGATCCGCACCGAGGTGGCCAATGACGAGCGACTGGTGGCCAAGCCGCTGCTGGCCTGGCAGGAAATGACCTGCGCCAGTCCTGAATATCTGGAGCGCTTTGGCGAACCGCTGACGCCGCAGGCG  CTGGCCGAGCACCGTTGCCTGCTCAACAGTCATTACAGCGGTCGCGAGGAATGGCTCTATCACCAGCAGCACGAACTGCTGCGGGTGCGGGTGTCGGGGCCGTTCGCCAGCAATCACTATAACCTGTTGAAGAAAGCCGCATTGGCCGGCGCCGGCATCGCTCGCCTGCCGTCCTACCTG  TTGCAGGCGGAATTGGCTGACGGCCGATTGCGCTGGCTCCTTCGCGATTTTCAGACCCGACGCATGCCGATGTATCTGGTGCACCCGTATCAGGGCGGTCTACCGAAACGCACCCAGGTGCTGGCAGATTACCTGATCGGCTGGTTCAAGCTCAGCGGCGAGGCGCTGGACCG |
| **CP000076.1** | *Pseudomonas protegens* Pf-5 | ATGGATGACCTGGCGGCATTTGCGGTGCTGGTGGAAGCCGGCAGCTTTACCTTGGCGGCCCAGCAGTTGGGGTGCAGCAAGGGCCAGCTCTCCAAACGCATCAGCGCCCTGGAAGCCCGCTACGCCGTGGTACTGCTGCAACGCACCACCCGGCGCCTGGACCTCACGGCCGCCGGCGCG  GCGTTGCTACCCCAGGCCCAGGCCCTGGTGGCTCAGGTCGAGCGGGCCCACCAGGCCCTGGCGCGGCTCAAGGACGATATGGTCGGGCCGGTGCGCCTCACCGTCCCGGTCTCCCTGGGGGAAACCTTTTTCGACGGCCTGCTGCTGGAGTTCTCCCGGCACTATCCCCAGGTGCAGATC  GAGCTGGACCTGAACAACAGCTACCGCGACCTGACCCGGGAGGGTTTCGATCTGGCCGTGCGCTCGGAGGTGGCCAACGACCAGCGCCTGGTGGCCCGGCCGCTGTTGGCCTGGCATGAAATGACCTGCGCCAGCCCGGCCTACCTGGAGCAATACGGCGAGCCGCGCACGCCTCAGGAA  CTGGCCGGGCATCGCTGCCTGCTCAATAGCCATTACAGCGGTCGGGAAGAGTGGTTGTACCACCAGCGCCATGAACTGTTGCGGGTGCGGGTCAGCGGCCCCTTCGCCAGCAACCACTACAACCTCCTGAAGAAGGCTGCCCTGGTGGGCGCGGGGATCGCCCGGCTGCCGTCCTATGTG  CTGCACAGCGAACTGGCGGACGGCCGCCTGCGCTGGCTGTTGCGGGACTACCAGACCCGCAGCATGCCGATGTACCTGGTGCACTCCTATCAGGGCGGCCTGCCCAAGCGTACCCAGGTGCTGGCCGATTACCTGATGGACTGGTTTCGTCGCAGCGGTGAGGCGCTGGATCG |
| **CP003190.1** | *Pseudomonas protegens* CHA0 | ATGGATGACCTGGCGGCATTTGCGGTGCTGGTGGAAGCCGGCAGCTTTACCCTGGCAGCCCAGCAGTTGGGGTGCAGCAAGGGCCAGCTCTCCAAACGCATCAGCGCCCTGGAAGCCCGCTACGCCGTGGTACTGCTGCAACGCACCACCCGACGTCTGGACCTGACCGCCGCCGGGGCG  GCGCTTCTGCCTCAGGCCCAGGCGCTGGTAGTCCAAGTGGAGCGGGCCCATCAGGCCCTGGCCCGGCTCAAGGACGATATGGCCGGCCCGGTGCGCCTGACGGTTCCGGTGTCCCTGGGAGAGACTTTTTTCGATGGCCTGCTGCTGGAGTTCTCCCGGCACTATCCCCAGGTGCAGATC  GAGCTGGACCTGAACAACAGCTACCGCGACCTGACCCGGGAGGGTTTCGATCTGGCCGTGCGCTCGGAGGTGGCCAACGACCAGCGCCTGGTGGCCCGGCCGCTGTTGGCCTGGCATGAAATGACCTGCGCCAGCCCGGCCTACCTGGAGCAATACGGCGAGCCGCGCACGCCTCAGGAG  CTGGCCGGGCATCGCTGCCTGCTCAATAGCCATTACAGCGGTCGGGAAGAGTGGTTGTACCACCAGCGCCATGAACTGTTGCGGGTGCGGGTCAGCGGCCCCTTCGCCAGCAACCACTACAACCTCCTGAAGAAGGCTGCCCTGGTGGGCGCGGGGATCGCCCGGCTACCGTCCTATGTG  CTGCACAGCGAACTGGCGGACGGCCGCCTGTGCTGGCTGTTGCGGGACTACCAGACCCGCAGCATGCCGATGTACCTGGTGCACTCCTATCAGGGCGGCCTGCCCAAGCGTACCCAGGTGCTGGCCGATTACCTGATGGACTGGTTTCGTCGCAGCGGTGAGGCGCTGGATCG |
| **CP012830.1** | *Pseudomonas fluorescens* strain FW300-N2E3 | ATGGATGATCTGGCGGCGTTTGCAGTGTTGATCGAGGCCGGCAGTTTCACGCTGGCAGCGCAGCAACTGGGGTGTAGCAAGGGGCAGTTGTCCAAGCGCATCAGTCAGCTGGAAACACGTTTCAGCGTAGTGCTGTTGCAGCGAACCACACGGCGTTTGAGCCTGACAGCCGCTGGTGCA  GCATTGTTGCCGCAAGCGCAGGCGCTGGTGATTCAGGTAGAGCGGGCGCGACAGGCCCTGGCACGCTTGAAGGACGACATGGCAGGGCCGATTCGGATGACGGTCCCGGTGTCGTTGGGGGAGACTTTTTTCGATGGGTTGCTGCTGGAGTTTTCCCGAGAGTACCCCGACGTGCAGATC  GAACTTGAGCTGAACAACAGTTACCGCGACTTGTCGCGGGACGGGTTCGATCTGGCGATTCGTTCAGAGGTGGCCAACGACGAGCGAATGGTCGCCAAACCACTGTTGGCCTGGCACGAGATGACCTGCGCCAGCCCGGCTTATCTTGAGCAGTTTGGTGAGCCGCAGACGCCGGCGGAC  CTGGCCGATCATCGTTGCCTGCTCAACAGCCATTACAGCGGCCGGGAAGAGTGGTTGTATCACCAGCAACATGAACTGTTGCGGGTGCGGGTCTCGGGACCGTTCGCCAGCAACCACTACAACCTGCTGAAGAAGGCTGCGCTTGCCCATGCGGGGATTGCGCGCCTGCCTTCTTACTTG  CTCCAGGCTGAATTGGCCGATGGCCGTTTGCGCTGGCTGCTGCGCGACTATCAGACCCGCAGCATGCCGATGTATCTGGTGCATCCGTATCAGGGCGGCTTGCCCAAGCGCACGCAGGTATTGGCCGACTACCTGATGAGGTGGTTCAAGCGCAGCGGCGAAGCACTGGATCGGCT |
| **LN854573.1** | *Pseudomonas* sp. URMO17WK12:I11 | AGGCGATCCAGCGCCTCACCGCTACGCTTGAACCAGCCGATCAGGTAATCCGCCAGCACCTGCGTGCGTTTCGGCAAACCGCCCTGATACGGGTGAACCAGGTACATCGGCATGCGCCGCGTCTGATAATCACGCAGGAGCCAGCGCAATCGCCCGTCAGCCAATTCCGTTTGCAGTAAA  TAGGACGGCAGGCGCGCAATGCCGGCCCCCGCCAATGCGGCTTTTTTCAGCAAGCTGTAGTGATTGCTGGCAAACGGCCCCGACACCCGTACGCGCAACAACTCATGTTGCTGGTGATAGAGCCATTCTTCGCGACCGCTGTAATGACTGTTGAGCAGGCAACGGTGTTCGGCCAGGGCC  TGCGGTGTTTGCGGTTCGCCATACTGTTCAAGATACGCCGGACTGGCGCAGGTCATCTCGTGCCAGGCCAGCAGTGGCCGAGCCACCAATCGCTCGTCCTTGCCCACCTCGGAACGAATCGCCAGATCGAAACCATCCCGAGACAGGTCGTGAAAGTTGTTGTTCAGATCGAGCTCGATC  TGCACCTCTGGGTACTTGCCGGAAAACTCCAGCAACAAACCATCGAAGAAGGTTTCCCCCAGCGATACCGGAACGGTCATACGCACCGGCCCGGCCATGTCGTCCTTCAACCGCGCCAATGCCTGACGCGCCCTTTCGACCTGGACGACAAGCGCCTGAGCTTGCGGCAACAGCGCCGCG  CCTGCCGCCGTCAGGCTTAAACGACGCGTCGTGCGTTGCAGCAGCACCACCGAAAACCGTGTTTCCAGCAAGCTGATGCGCTTGGACAATTGGCCTTTGCTGCAACCCAGTTGCTGCGCTGCCAAGGTAAAACTGCCGGCTTCGATCAAGACCGCGAACGCCGCCAGGTCATCCAT |
| **CP005960.1** | *Pseudomonas mandelii* JR-1 | GAGACGCGCCAATGCCTCGCCGCTGCGTTTAAACCAATCAATCAAGTAGTCCGCCAACACCTGCGTGCGTTTGGGCAGTCCGCCCTGATACGGATGCACCAGGTACATCGGCATGCTCCGCGTCTGATAATCACGCAGGAGCCAGCGCAATCGCCCGTCAGCCAATTCCGTTTGCAGCAG  GTAGGAAGGCAACCGCGCAATGCCGGCCCCTGCCAGCGCTGCTTTTTTCAACAGGCTGTAGTGATTGCTGGCAAACGGACCCGACACTCGCACCCGCAGCAACTCGTGCTGCTGGTGATACAGCCATTCTTCGCGACCGCTGTAATGGCTGTTGAGCAGACAGCGATGGTCGGCCAACGC  CTGGGGCGTCTGCGGCTCACCGTACTGTTCGAGATAGGCCGGGCTGGCGCAGGTCATTTCGTGCCAGGCCAGCAGAGGACGCGCGACCAAGCGTTGGTCATTGGCCACTTCGGAGCGGATGGCCAGGTCAAAACCGTCTCGGGACAAATCGTGAAAGCTGTTGTTGAGGTCCAACTCGAT  CTGCACCTCGGGATACTGGCGGGAAAATTCCAGCAACAAGCCATCGAAGAAGGTTTCCCCCAGCGAGACCGGAACCGTCATGCGCACCGGCCCGGCCATGTCGTCCTTCAACCGCGCCAACGCCTGACGCGCCTTCTCGACTTGAACCACCAGCGCCTGGGCTTGAGGCAACAGCGCCGC  GCCCGCCGCCGTCAAACTCAAACGGCGGGTGGTGCGTTGCAGCAGGACCACAGAGAACCGTGTTTCCAGCAAGCTGATGCGCTTGGACAACTGTCCCTTGCTGCAGCCCAATTGCTGCGCCGCCAGGGTGAAACTGCCCGCTTCGATCAACACCGCGAACGCTGCCAGATCATCCAT |
| **CP003150.1** | *Pseudomonas fluorescens* F113 | ATGGATGACCTGGCGGCGTTTGCCGTGTTGATGGAGGCCGGCAGCTTCACCCTGGCGGCCCAGCAGTTGGGGTGCAGCAAGGGCCAGTTGTCCAAGCGCATCAGCCTGTTGGAGAGTCGGTTTTCGGTGGTGTTGCTGCAACGCACCACGCGGCGCTTGAGCCTTACGGCGGCAGGGGCG  GCGTTGTTGCCCCAGGCCCAGGCGCTGTTGGTGCAGGTGGAACGGGCACGTCAGGCATTGGCGCGTTTGAAGGACGATATTTCTGGGCCGGTGCGTATGACGGTTCCGGTTTCGTTGGGTGAAACGTTCTTCGAGGGCTTGCTGATGGAGTTCGCTCGTACGTATCCCCACGTACAGATC  GAACTGGAGCTCAACAACGGTTACCGCGACCTGACCCGCGATGGTTTCGACCTGGCGATCCGTTCCGACGCCGCTATCGATGAGCGGCTGGTGGCCCGGCCATTGCTGGCATGGCACGAAATGACCTGCGCCAGTCCGGCCTACCTCGAACTCTATGGCGAACCCGAGACGCCCCAGGCC  CTGGCCGAACATCGCTGCCTGCTCAACAGCCACTACAGCGGCCGGGAAGAATGGCTTTACCACCAGCAACACGAGCTGCTGCGGGTCCGAGTCTCGGGGCCGTTCGCCAGCAACCACTACAGCCTGTTGAAAAAAGCCGCCCTGGCCGGCGCCGGAATCGCCCGGCTGCCGTCGTATTTG  CTGCATGAGGAGTTGGCCGACGGACGCCTGCACTGGCTGCTGCGCGACTACCAGACCCGGCGCATGCCGATGTACCTGGTGCACCCGTATCAGGGTGGATTGCCCAAGCGCACCCAGGTGCTCGCCGACTACTTGATCGACTGGTTCAAGCGCAGCGGCGAGGCGTTGGATCGGCT |
| **CP012680.1** | *Pseudomonas brassicacearum* strain LBUM300 | ATGGATGACCTGGCGGCGTTCGCCGTGTTGATGGAGGCCGGCAGCTTCACCCAGGCGGCCCAGCAGTTAGGCTGCAGCAAGGGCCAGTTGTCCAAGCGCATCAGTCTGTTGGAGAGGCGGTTTTCGGTGGTGTTGCTGCAACGCACCACGCGGCGCCTGAGCCTCACGGCGGCAGGGGCG  GCGTTGCTGCCCCAGGCCCAGGCGCTGCTGGTGCAGGTAGAAAGGGCGCGTCAGGCATTGGCCCGGTTGAAGGACGATATTTCCGGACCGGTGCGTATGACGGTTCCGGTTTCGTTGGGCGAAACGTTCTTCGAAGGCTTGCTGATGGAGTTCGCTCGTACGTATCCCAACGTGCAGATC  GAGCTGGAGCTCAACAACGGTTACCGTGACCTGACCCGCGATGGTTTCGATCTGGCGATCCGTTCCGACGCCGCCATCGACGAGCGGCTGGTGGCCCGGCCATTGTTGGCGTGGCACGAAATGACCTGCGCCAGCCCGGCCTACCTGGAACGCTATGGCGAACCTGAGACCCCTCAGGCC  CTGGCTGAGCATCGCTGCCTGCTCAACAGCCACTACAGCGGCCGGGAAGAATGGCTTTACCACCAGCAACACGAACTGCTGCGGGTCCGAGTGTCGGGGCCGTTCGCCAGTAACCACTACAGCCTGCTGAAAAAAGCCGCCTTGGCCGGCGCCGGAATCGCCCGGCTGCCGTCGTACCTG  CTGCATGAGGAGTTGGCCGACGGACGCCTGCACTGGCTGCTGCGCGACTACCAGACCCGGCGCATGCCGATGTACCTGGTGCACCCGTACCAGGGTGGATTGCCCAAGCGCACCCAGGTGCTGGCTGACTATCTGATCGACTGGTTCAAGCGCAGCGGCGAGGCGTTGGATCGGCT |
| **CP012831.1** | *Pseudomonas fluorescens* strain FW300-N2C3 | AGCCGATCCAACGCCTCGCCACTGCGCTTGAACCAGTCGATCAAATAGTCGGCCAGCACCTGGGTGCGCTTGGGCAGGCCACCCTGGTACGGGTGCACCAGGTACATCGGCATGCGCCGGGTCTGGTAGTCGCGCAGCAGCCAATGCAGGCGTCCATCCGCCAACGCTTCATGCAGCAGG  TACGACGGCAGCCGGGCGATTCCGGCACCGGCCAGGGCGGCTTTTTTCAGCAGGCTGTAGTGATTGCTGGCGAACGGCCCCGACACTCGGACCCGCAACAGTTCGTGTTGTTGGTGGTAAAGCCATTCTTCCCGGCCGCTGTAGTGGCTGTTGAGCAAACAGCGGTGCTCGGCCAGGGCC  TGGGGCGTCTCAGGCTCGCCGTAGTGTTCGAGGTAGGCCGGGCTGGCGCAGGTCATTTCGTGCCACGCCAACAATGGCCGGGCCACCAGCCGCTCATCGATGGCGGCGTCGGAGCGGATCGCCAGGTCGAAACCATCGCGGCTCAAGTCACGGTAACCGTTGTTGAGCTCCAGTTCGATC  TGCACATTGGGGTACGTGCGAGCGAACTCCATCAGCAAGCCCTCGAAGAAGGTTTCACCCAACGAAACCGGAACCGTCATACGCACCGGTCCGGAAATGTCATCCTTCAACCGAGCCAATGCCTGACGCGCCCTTTCCACCTGCACCAGCAGCGCCTGGGCCTGAGGCAACAGCGCCGCC  CCTGCCGCCGTGAGGCTCAAGCGCCGTGTGGTGCGTTGCAGCAACACCACCGAAAACCGGCTCTCCAACAGGCTGATGCGCTTGGACAACTGGCCCTTGCTGCACCCCAATTGCTGGGCCGCCAAGGTAAAACTGCCGGCCTCCATCAACACGGCGAACGCCGCCAGGTCATCCAT |
| **CP002585.1** | Pseudomonas brassicacearum subsp. brassicacearum NFM421 | ATGGATGACCTGGCGGCGTTCGCCGTGTTGATGAAGGCCGGCAGCTTCACCCAGGCGGCCCAGCAGTTAGGCTGCAGCAAGGGCCAGTTGTCCAAGCGCATCAGTCTGTTGGAGAGGCGGTTTTCGGTGGTGTTGCTGCAACGCACCACGCGGCGCCTGAGCCTCACGGCGGCAGGGGCG  GCGTTGCTGCCCCAGGCCCAGGCGCTGCTGGTGCAGGTAGAAAGGGCGCGTCAGGCATTGGCCCGGTTGAAGGACGATATTTCCGGACCGGTGCGTATGACGGTTCCGGTTTCGTTGGGCGAAACGTTCTTCGAAGGCTTGCTGATGGAGTTCGCTCGTACGTATCCCAACGTGCAGATC  GAGCTGGAGCTCAACAACGGTTACCGTGACCTGACCCGCGATGGTTTCGATCTGGCGATCCGTTCCGACGCCGCCATCGACGAGCGGCTGGTGGCCCGGCCATTGTTGGCGTGGCACGAAATGACCTGCGCCAGCCCGGCCTACCTGGAACGCTATGGCGAACCTGAGACCCCTCAGGCC  CTGGCTGAGCATCGCTGCCTGCTCAACAGCCACTACAGCGGCCGGGAAGAATGGCTTTACCACCAGCAACACGAACTGCTGCGGGTCCGAGTGTCGGGGCCGTTCGCCAGTAACCACTACAGCCTGCTGAAAAAAGCCGCCTTGGCCGGCGCCGGAATCGCCCGGCTGCCGTCGTACCTG  CTGCATGAGGAGTTGGCCGACGGACGCCTGCACTGGCTGCTGCGCGACTACCAGACCCGGCGCATGCCGATGTACCTGGTGCACCCGTACCAGGGTGGATTGCCCAAGCGCACCCAGGTGCTGGCTGACTATCTGATCGACTGGTTCAAGCGCAGCGGCGAGGCGTTGGATCGGCT |
| **AM181176.4** | *Pseudomonas fluorescens* SBW25 | ATGGATGATCTGGCGGCCTTTGCCGTACTGATAGAAGCCGGTAGTTTCACCCTGGCCGCCGAGCAACTGGGCTGCAGCAAAGGGCAGCTGTCCAAGCGGATCAGCCAGTTGGAGGCGCAGTTTTCCGTGGTATTGCTGCATCGCACCACGCGCAAACTCAGCCTGACTGCTGCGGGTGCG  GCATTGCTGCCCCAGGCCCAGGCATTGGTGGTGCAAGTGGATCGCGCCCGCCAGGCACTGGCTCGGCTTAAGGATGACCTGGCCGGGCCTGTGCGTATGACGGTTCCGGTGTCGCTGGGCGAAACCTTTTTCGATGGCTTGTTACTGGAGTTTTCCAAACAGTATCCGCAGGTGCAGATC  GAGCTGGAGCTCAACAACCGCTACCGTGATCTGGCCCGGGACGGCTTTGACCTGGGCGTACGCTCAGGCGCGATTGAAAACGAGCGCCTGGTGGCCAAGCCGTTGCTGGCCTGGCATGAGATGACCTGCGCCAGCCCGGCCTACCTCGAGCACTATGGTGAACCCGAGACGCCGGCTGAC  CTGGCCGCGCATATCTGCCTGCTCAACAGTCACTACAGCGGTCGCGAGGAGTGGTTGTACCACCAGCAGCACGAATTGTTGCGGGTGCGGGTCAGTGGCACCTTTGCCTCCAATCACTACAACCTGTTGAAAAAGGCCGCGCTGGTGGGCGCGGGTATCGCGCGCCTGCCATCCTATGTA  CTGCCGGCGGAATTGGCTGACGGCCGATTGCGTTGGCTCCTGCGCGATTATCAGACGCGGAGCATGCCGATGTACCTGGTGCACCCCTATCAGGGCGGCCTGCCGCGTCGCACCCAGGTGTTGGCCGACTACCTGGTGGATTGGTTCAAGCGCAGTGGCGAGGCGCTGGATCGGCT |
| **CP007637.1** | *Pseudomonas simiae* strain | ATGGATGACCTGGCCGCGTTTGCGGTGCTGATCGAAGCCGGTAGTTTTACCGTGGCCGCCGAGCAATTGGGCTGCAGCAAAGGGCAGTTGTCCAAGCGGATCAGCCAGTTGGAAGCGCGGTTTTCCGTGGTATTGCTGCACCGCACCACCCGCAAGCTTAGCCTGACCGCGGCCGGCGCA  GCGTTGCTGCCCCAGGCCCAGGCATTGGTGGTGCAGGTGGATCGTGCCCGACAGGCGTTGGCCCGGCTCAAGGATGATCTGGCTGGGCCGGTACGCATGACGGTGCCGGTGTCGCTGGGCGAGACCTTCTTCGACGGATTGTTGCTGGAGTTTTCCAAGCAGTACCCGCAGGTACAGATC  GAGCTGGAGCTCAACAACAGCTACCGCGACCTGGCGCGGGATGGCTTTGACCTGGGGGTACGCTTAGGCGCGATCGAGAATGAGCGCCTGGTGGCCAAGCCGTTGTTGGCCTGGCATGAGATGACCTGCGCCAGCCCAGCCTACCTTGAGGAGCATGGCGAGCCGCAGACCCCGTCCGAC  CTGGCCGCGCACACCTGCCTGCTCAACAGCCATTACAGCGGGCGTGAAGAGTGGCTGTATCACCAGCAGCACGAATTGCTGCGGGTGCGGGTCAGCGGCACATTCGCCTCCAACCACTACAACCTGTTGAAAAAGGCCGCATTGGTGGGCGCCGGTATTGCGCGGCTGCCGTCCTACGTT  CTCCCCGCGGAATTAGCGGACGGCCGTTTGCGCTGGCTCCTGCGCGATTATCAGACGCGGAGCATGCCGATGTACCTGGTGCATCCCTATCAGGGCGGTTTACCGCGCCGCACCCAGGTGTTGGCCGATTATCTGGTGGGTTGGTTCAAGCGCAGCGGCGAGGCCCTGGATCGGCT |
| **CP005975.1** | *Pseudomonas fluorescens* PICF7 | AGCCGATCCAGGGCCTCGCCGCTGCGCTTGAACCAACCCACCAGATAATCGGCTAACACCTGGGTGCGGCGCGGCAAACCGCCCTGATAGGGATGCACCAGGTACATCGGCATGCTCCGCGTCTGATAATCGCGCAGGAGCCAGCGCAAACGGCCGTCCGCTAATTCCGCGGGGAGAACG  TAGGACGGCAGCCGCGCAATACCGGCGCCCACCAATGCGGCCTTTTTCAACAGGTTGTAGTGGTTGGAGGCGAATGTGCCGCTGACCCGCACCCGCAGCAATTCGTGCTGCTGGTGATACAGCCACTCTTCACGCCCGCTGTAATGGCTGTTGAGCAGGCAGGTGTGCGCGGCCAGGTCG  GACGGGGTCTGCGGCTCGCCATGCTCCTCAAGGTAGGCTGGGCTGGCGCAGGTCATCTCATGCCAGGCCAACAACGGCTTGGCCACCAGGCGCTCATTCTCGATCGCGCCTAAGCGTACCCCCAGGTCAAAGCCATCCCGCGCCAGGTCGCGGTAGCTGTTGTTGAGCTCCAGCTCGATC  TGTACCTGCGGGTACTGCTTGGAAAACTCCAGCAACAATCCGTCGAAGAAGGTCTCGCCCAGCGACACCGGCACCGTCATGCGTACCGGCCCAGCCAGATCATCCTTGAGCCGGGCCAACGCCTGTCGGGCACGATCCACCTGCACCACCAATGCGTGGGCCTGGGGCAGCAACGCTGCG  CCGGCCGCGGTCAGGCTAAGCTTGCGGGTGGTGCGGTGCAGCAATACCACGGAAAACCGCGCTTCCAACTGGCTGATCCGCTTGGACAACTGCCCTTTGCTGCAGCCCAATTGCTCGGCGGCCACGGTAAAACTACCGGCTTCGATCAGCACCGCAAACGCGGCCAGGTCATCCAT |
| **CP010896.1** | *Pseudomonas fluorescens* strain PCL1751 | ATGGATGACCTGGCCGCGTTTGCGGTGCTGATCGAAGCCGGTAGTTTTACCGTGGCCGCCGAGCAATTGGGCTGCAGCAAAGGGCAGTTGTCCAAGCGGATCAGCCAGTTGGAAGCGCGGTTTTCCGTGGTATTGCTGCACCGCACCACCCGCAAGCTTAGCCTGACCGCGGCCGGCGCA  GCGTTGCTGCCCCAGGCCCAGGCATTGGTGGTGCAGGTGGATCGTGCCCGACAGGCGTTGGCCCGGCTCAAGGATGATCTGGCTGGGCCGGTACGCATGACGGTGCCGGTGTCGCTGGGCGAGACCTTCTTCGACGGATTGTTGCTGGAGTTTTCCAAGCAGTACCCGCAGGTACAGATC  GAGCTGGAGCTCAACAACAGCTACCGCGACCTGGCGCGGGATGGCTTTGACCTGGGGGTACGCTTAGGCGCGATCGAGAATGAGCGCCTGGTGGCCAAGCCGTTGTTGGCCTGGCATGAGATGACCTGCGCCAGCCCAGCCTACCTTGAGGAGCATGGCGAGCCGCAGACCCCGTCCGAC  CTGGCCGCGCACACCTGCCTGCTCAACAGCCATTACAGCGGGCGTGAAGAGTGGCTGTATCACCAGCAGCACGAATTGCTGCGGGTGCGGGTCAGCGGCACATTCGCCTCCAACCACTACAACCTGTTGAAAAAGGCCGCATTGGTGGGCGCCGGTATTGCGCGGCTGCCGTCCTATGTT  CTCCCCGCGGAATTAGCGGACGGCCGTTTGCGCTGGCTCCTGCGCGATTATCAGACGCGGAGCATGCCGATGTACCTGGTGCATCCCTATCAGGGCGGTTTGCCGCGCCGCACCCAGGTGTTAGCCGATTATCTGGTGGGTTGGTTCAAGCGCAGCGGCGAGGCCCTGGATCGGCT |
| **CP006852.1** | *Pseudomonas* sp. TKP | ATGGATGACCTGGCCGCCTTTGCGGTGTTGATCGAGGCCGGCAGTTTCACCCTGGCGGCGGAGCAGTTGGGTTGCAGCAAGGGGCAACTGTCCAAGCGCATCAGCCAGTTGGAAGCGCAGTTTGCGGTGATATTGCTGCACCGCACCACGCGCAAGCTGAGCCTCACTGCCGCCGGCGCA  GCGTTGTTGCCCCAGGCCCAGGCATTGGTGGTACAGGTGGATCGCGCCCGCCAGGCGCTGGCCCGGCTCAAGGACGATCTGTCGGGGCCTGTGCGTATGACGGTGCCGGTGTCGTTGGGCGAAACGTTCTTCGATGGCCTGTTATTGGAGTTTTCCAAGCAGTACCCACAGGTGCAGATT  GAGCTGGAACTCAACAATAGCTACCGGGATTTGGCGCGGGACGGTTTTGACCTGGGCGTGCGCTCGGGGGCGATCGAACATGAGCGCCTGGTGGCCAAGCCGCTGCTGGCCTGGCATGAGATGACCTGCGCGAGCCCGGCCTACCTGGAGCAGCACGGCGAACCCCTGACCCCGGCGGAC  CTGGCCGCGCACAGCTGTTTGCTCAACAGTCACTACAGTGGCCGTGAAGAGTGGTTGTACCACCAGCAGCACGAATTGCTGCGGGTCCGCGTGAGCGGCACCTTTGCCTCCAATCATTACAACCTGCTGAAAAAGGCCGCACTGGTGGGGGCCGGTATCGCCCGCCTGCCGTCCTATGTA  TTACCCGCGGAACTGGCTGACGGCCGATTGCGCTGGCTCCTGCGCGATTATCAGACACGCAGCCTGCCGATGTACCTGGTCCACCCCTACCAAGGTGGCCTGCCACGCCGAACCCAGGTCTTGGCCGACTACCTGGTGGACTGGTTCAAGCGCAGTGGCGAGGCCCTGGACCGCCTCT |
| **CP011020.1** | *Pseudomonas chlororaphis* strain UFB2 | GAGACGGTCCAACGCTTCACCGCTGCGCTTGAACCAGTCGATCAGATAGTCGGCCAGCACCTGCGTCCGCTTGGGCAGGCCACCTTGATAGGGATGCACCAAGTACATCGGCATGCGCCGAGTCTGAAAACCGCTCAGGATCCGTCGCAAGCGCCCGTCAGCCAATTCTTCGTGCAACAG  GTACGACGGCAGCCGGGCGATTCCGGCACCGGCCAGGGCGGCTTTTTTCAACAGGCTGTAGTGGTTGCTGGCGAACGGCCCCGACACCCGGACCCGCAGCAGTTCGTGCTGCTGGTGGTACAACCATTCCTCCCGGCCGCTGTAATGGCTGTTGAGCAGGCAGCGGTGCTCCGCCAGGGC  CTGGGGCGTGTCCGGTTCGCCATGACGGTCGAGATAAGCCGGGCTGGCGCAGGTCATTTCGTGCCACGCCAACAACGGCCGAGCCACCAGTCGCTCGTCGATGGCGGCGTCCGAGCGGATCGCCAGGTCGAAGCCTTCGCGGGCCAGGTCGCGGTAGCCGTTGCTGAGTTCCAGTTCAAT  CTGCACATGGGGATACGTGTGAGCGAACTCCACCAGCAAACCCTCAAAAAAGGTTTCGCCCAACGACACCGGAACTGTCATACGCACCGGCCCGGCCACGTCGTCCTTCAATCGGGCCAATGCCTGACGCGCCTTTTCCACCTGCACCAGCAGCGCCTGGGCCTGGGGCAACAACGCCGC  CCCCGCCGCCGTGAGGCTCAGGCGCCGGGTGGTGCGTTGCAGCAGCACCACGCTGAAGCGGCTTTCCAACAGGCTGATGCGCTTGGACAACTGGCCCTTGCTACAGCCCAACTGTTGCGCCGCCAGAGTAAAGCTGCCGGCCTCCATCAATACGGCAAACGCCGCCAGGTCATCCAT |
| **CP007410.1** | *Pseudomonas brassicacearum* strain DF41 | ATGGATGATCTGGCGGCATTTGCCGTCTTGATGGAGGCCGGCAGCTTCACCCTGGCGGCCCAGCAGTTAGGGTGCAGCAAGGGGCAGCTGTCCAAGCGCATCAGCCTGTTGGAGAGCCGCTTTTGCGTGGTGTTGCTGCAACGCACCACGCGGCGCTTGAGCCTCACGGCCGCCGGCGCG  GCGTTGTTGCCCCAGGCCCAGGCGTTGCTGGTGCAGGTGGAAAGGGCGCGTCAGGCATTGGCTCGGTTGAAGGACGATATTTCCGGGCCGGTACGTATGACAGTTCCGGTTTCGTTGGGCGAAACCTTCTTTGAAGGTTTGCTGCTGGAGTTCGCTGGCACGTACCCCGACGTGCAAATC  GAGCTGGAGCTCAACAACGGCTACCGCGACCTGACCCGCGACGGCTTCGATCTGGCGATCCGTTCCGACGCCGCTATCGATGAGCGATTGGTGGCCCGGCCGCTGTTGGCGTGGCATGAAATGACCTGCGCCAGCCCGGCCTATCTCGAAGGCTATGGCGAACCTGAAAACCCCAAGGCC  CTGGCGGAACACCGTTGCCTTCTCAACAGCCACTACAGCGGTCGCGAAGAATGGCTGTATCACCAGCAACACGAACTGTTGCGCGTGCGGGTGTCGGGGCCGTTCGCCAGTAATCATTACAGCCTGTTGAAAAAAGCCGCCCTGGCTGGTGCCGGCATCGCCCGGTTGCCGTCGTACCTG  CTGCATGAAGAATTGGCTGACGGACGCCTGCGCTGGTTGCTGCGCGATTACCAGACCCGTCGCATGCCGATGTACCTGGTGCACCCGTATCAAGGTGGGTTGCCCAAGCGCACTCAGGTGTTGGCCGATTACTTGATCGATTGGTTCAAGCGCAGCGGCGAGGCGTTGGATCGC |
| **CP008896.1** | *Pseudomonas fluorescens* strain UK4 | ATGGATGATTTGGCGGCGTTTGCGGTGTTGATCGAAGCCGGTAGCTTTACCTTGGCGGCCCAGCAGCTGGGATGCAGCAAGGGCCAGTTGTCCAAGCGCATCAGCCAGTTGGAGGCGCAATTTTCGGTAGTCTTGTTGCATCGCACCACTCGACGACTCAGTCTGACGGCCGCCGGGGCG  GCGTTATTGCCTCAAGCCCAGGGGCTGGTGATCCAGGCCGAGCGTGCCCGACAGGCATTGGCGCGGCTCAAGGACGATTTGGCTGGGCCAGTGCGTATGACGGTTCCGGTGTCTTTGGGAGAAACCTTCTTCGACGGTCTTTTGTTGGAGTTTTCCGGGCGGTATCCGCAGGTGCAGATC  GAGTTGGACCTCAGCAATAGTTACCGCGACCTGGCCAGGGACGGTTTTGACTTGGCGGTGCGCTCAGAGGTCGCCAACGATGAGCGTCTGGTTGCCAAGCCCCTGTTGGCATGGCATGAAATGACCTGCGCCAGCCCGGCCTACCTGGAGCAATACGGTGAGCCGCTGACACCGTCGGAA  CTGGTTGGCCACCGCTGTTTGCTCAACAGCCACTACAGCGGGCGCGAAGAGTGGCTGTATCACCAGCAACACGAGTTGTTGAGGGTACGAGTGGATGGCACCTTTGCCTCCAATCACTACAACCTGCTGAAAAAGGCTGCGCTGGTGGGGGCCGGTATTGCCCGGCTGCCATCCTATCTG  TTGCAAATGGAGCTGGCTGACGGCCGGCTGCGCTGGCTCCTGCGTGAATATCAGACTCGCAGTATGCCGATGTATCTGGTTCATCCCTATCAGGGCGGCCTGCCACGTCGTACCCAAGTGCTGGCCGACTACCTGGTCGGCTGGTTCAAGCGCAACGGCGAGGCGTTGGACCGGCT |
| **ANOR01000035.1** | *Pseudomonas fluorescens* Pf29Arp | AGCCGATCCAACGCCTCCCCACTCCTCTTGAACCACTCAATCAGATAATCAGCCAGCACCTGCGTGCGCTTGGGCAGCCCACCCTGATACGGGTGCACCAGGTACATCGGCATGCGCCGGGTCTGGAAGTCGCGCAGCAACCAACGCAGACGTCCGTCAGCCAATTCCTCGTTGAGCAGG  TAAGACGGTAACCGGGCGATGCCGGCACCCGCCAGGGCGGCTTTTTTCAACAGGCTGTAATGGTTGCTGGCAAACGGCCCGGACACTCGCACCCGCAGCAATTCGTGTTGCTGGTGGTACAGCCATTCCTCACGTCCGCTGTAGTGGCTGTTGAGCAAGCACTTGTGCTCCGCCAGGGCC  TGGGGTGTTTCCGGCTCGCCGTAGCGCTCCAGATAAGCCGGGCTGGCGCAGGTCATTTCATGCCAGGCCAGCAACGGACGAGCCACCAATCGCTCATCGATCGCCGCATCGGAGCGGATCGCCAGGTCGAAACCATCGCGGGATAGATCGCGATAGCCGTTGTTGAGCTCCAGCTCGATC  TGCACGTCGGGATAGGCATGGGCAAACTCGGTAAGCAGCCCCTCGAAGAAGGTTTCGCCCAACGAAACCGGAACCGTCATACGCACCGGCCCGGAAACTTCGCCCTTCAAGCGGGCCAATGCCTGACGCGCCCTTTCCACCTGGACCAACAACGCCTGGGCCTGGGGCAACAGCGCAGCG  CCCGCCGCAGTGAGACTCAAGCGTCGTGTCGTGCGCTGGAGCAGCAGCACCGAAAACCGTTCTTCCAGAAGACTGATGCGCTTGGACAGTTGCCCCTTGCTGCACCCCAACTGCTGCGCCGCCAAGGTGAAACTTCCAGCCTCCACCAGCACGGCAAACGCGGCGAGGTCGTCTAT |
| **CP003041.1** | *Pseudomonas fluorescens* A506 | ATGGATGACTTGGCGGCGTTCGCCGTGCTGATCGAAGCCGGCAGTTTCACTGTGGCGGCCTCACAATTGGGCTGCAGCAAAGGGCAGTTGTCCAAGCGCATCAGCCAGTTGGAAGCGCAGTTTGCCGTGGTTTTGCTGCACCGCACCACGCGCAAGCTGAGCCTTACTGCTGCCGGCGCG  GCGTTGCTGCCCCAGGCACAAGCATTGGTGGTGCAGGTGGAACGCGCGCGCCAGGCCTTGGCGCGGCTCAAAGATGATTTGGCCGGCCCTGTACGTATGACTGTCCCGGTGTCGTTGGGTGAAACCTTTTTCGATGGCTTGTTGCTGGAATTTTCCAAGCAATACCCGCAGGTGCAGATC  GAGCTGGAGCTTAACAACAGCTACCGGGACCTGGCGCGCGATGGCTTTGACTTGGGCGTTCGCTCGGGCGCCCTCGAAAATGAACGCCTGGTAGCCAGGCCTTTACTGGCCTGGCATGAGATGACCTGCGCCAGCCCGGCCTATCTGGAACAGCATGGCGAGCCGCAGACCCCGGCAGAC  CTGGCGGCGCACAGCTGTTTGCTCAACAGTCATTACAGTGGTCGCGAGGAATGGTTGTACCACCAGCAACACGAATTGCTGCGGGTAAAGGTGAGCGGCACCTTCGCCTCCAACCACTACAACCTGCTGAAAAAAGCCGCCTTGGTCGGCGCCGGTATTGCTCGCCTGCCGTCCTACGTT  TTACCCACGGAGCTGGCTGATGGCCGATTGCGCTGGCTGCTGCGCGACTATCAGACGCGCAGCATGCCGATGTACCTGGTGCATCCCTACCAAGGCGGCCTGCCACGGCGTACCCAAGTGTTGGCCGACTACCTGGTGGACTGGTTCAAGCGCAGCGGCGAGGCACTGGATCGACT |
| **CP007638.1** | *Pseudomonas* sp. WCS374 | ATGGATGACTTGGCGGCGTTCGCCGTGCTGATCGAAGCCGGCAGTTTCACTGTGGCGGCCGCACAATTGGGCTGCAGCAAAGGGCAGTTGTCCAAGCGCATCAGCCAGTTGGAAGCGCAGTTTGCCGTGGTTTTGCTGCACCGCACTACGCGCAAGCTGAGCCTTACTGCTGCCGGCGCG  GCGTTGCTGCCCCAGGCACAAGCATTGGTGGTGCAGGTGGAACGCGCGCGCCAGGCCTTGGCGCGGCTCAAAGATGATTTGGCCGGCCCTGTACGTATGACTGTCCCGGTGTCGTTGGGTGAAACCTTTTTCGATGGCTTGTTGCTGGAATTTTCCAAGCAATACCCGCAGGTGCAGATC  GAGCTGGAGCTTAACAACAGCTACCGGGACCTGGCGCGCGATGGCTTTGACTTGGGCGTTCGCTCGGGCGCCCTCGAGAATGAACGCCTGGTAGCCAGGCCTTTACTGGCCTGGCATGAGATGACCTGCGCCAGCCCGGCCTATCTGGAACAGCATGGCGAGCCGCAGACCCCGGCGGAC  CTGGCGGCGCACAGCTGTTTGCTCAACAGTCATTACAGTGGCCGCGAGGAATGGTTGTACCACCAGCAACACGAATTGCTGCGGGTAAAGGTGAGCGGCACCTTCGCCTCCAACCACTACAACCTGCTGAAAAAAGCCGCCTTGGTCGGCGCCGGTATTGCTCGCCTGCCGTCCTACGTT  TTACCCACGGAGCTGGCTGATGGCCGATTGCGCTGGCTGCTGCGCGACTATCAGACGCGCAGCATGCCGATGTACCTGGTGCATCCCTACCAAGGCGGCCTGCCACGGCGTACCCAAGTGTTGGCCGACTACCTGGTGGACTGGTTCAAGCGCAGCGGCGAGGCACTGGATCGACT |
| **CP011507.1** | *Pseudomonas trivialis* strain IHBB745 | AGAGTCGGTCGAGGGCCTCGCCGCTGCGCTTGAACCAGTCCACCAAGTAGTCAGCCAACACCTGGGTACGACGCGGCAGTCCGCCCTGATAGGGATGCACCAGGTACATCGGCATGCTCCGCGTCTGATAATCACGCAGGAGCCAACGCAAGCGGCCGTCAGCCAATTCCGCGGGTAGAA  CGTAGGACGGCAGGCGCGCGATACCAGCGCCCACTAACGCAGCCTTTTTCAACAAGTTGTAGTGATTGGAGGCAAAGGTTCCGCTGACACGCACCCGCAGCAGTTCGTGCTGCTGGTGGTACAACCACTCTTCACGACCGCTGTAGTGGCTGTTGAGCAGGCAGGTGTGGGCGGCCAAGT  CGGCCGGTGTCTCCGGCTCGCCATGCCTCTCAAGGTACGCCGGGCTGGCGCAGGTCATTTCATGCCAGGCCAGCAGAGGCTTGACCACCAGGCGCTCGTTTTCAATCGCGCCTGAGCGTACCCCCAAGTCAAAGCCATCCCGCGCCAGGTCCCGGTAGCTGTTGTTGAGCTCCAGCTCGA  TCTGCACCTGCGGGTACTGTTTGGAAAACTCCAGCAACAAGCCATCAAAGAAGGTTTCGCCCAGGGAGACCGGAACCGTCATACGCACCGGCCCGGCCAGATCGTCCTTTAGCCTGGCCAACGCCTGACGGGCGCGATCGACCTGCACCACCAATGCCTGAGCCTGGGGCAGCAATGCCG  CACCGGCTGCAGTCAGGCTGAGCTTGCGCGTCGTTCGGTGCAGCAGCACCACAGCATGCTGTGCTTCCAACTGGCTGATTCGCTTGGACAACTGCCCTTTGCTACAGCCCAGTTTCTCCGCGGCCACGGTAAAACTGCCGGCGTCGATCAGCACGGCGAAGGCTGCCAAATCATCCAT |
| **CP004045.1** | *Pseudomonas poae* RE*1-1-14 | ATGGATGATCTGGCGGCCTTTGCCGTGTTGATCGAAGCGGGCAGTTTTACCGTGGCGGCTGCGCGTCTGGGCTGTAGCAAGGGGCAGTTGTCAAAGCGCATCAGCCAGTTGGAAGCACAGTTTGATGTGGTGCTGCTGCACCGTACCACGCGCAAACTGAGCCTCACCGCCGCAGGTGTA  GCCTTGTTGCCCCAGGCGCAGGCGTTGGTGGTGCAGATGGAATGCGCGCGTCAGGCATTGGCGCGGCTCAAGGACGATGTGGCCGGGCCGGTGCGCATGACGGTTCCGGTTTCATTGGGTGAAACCTTCTTCGACGGCCTGTTGCTGGACTTTTCCAGGCAATACCCGCAGGTGCAGGTC  GAGTTGGAGCTTAACAACAGCTACCGCGATCTGGCGCGGGACGGGTTTGACCTGGGGGTGCGCGCGGGCGACATCGAACATGAGCGGCTCGTGGCCAGGCCCTTGCTGGCCTGGCACGAGATGACCTGCGCCAGCCCCGCCTACCTCGAACAGCACGGTGAACCGCAGACCCCGGCGGAT  CTGGCCGCCCACACCTGTTTGCTGAACAGCCACTACCGCGGCCGCGAGGAGTGGCTGTACCACCAGCAGCACGAATTATTGCGGGTGCGCGTGAGCGGCACGTTTGCCTCCAACCATTACAACCTGCTGAAGAAGGCCGCGCTGGTCGGCGCCGGTATCGCCCGTCTGCCGTCCTACGCC  TTGCCAACGGAACTGGCTGACGGACGACTGCGCTGGCTCCTGCGTGATTATCAGACGCGGAGCATGCCGATGTACCTGGTTCACCCTTACCAGGGCGGCTTGCCACGACGCACCCAGGTATTGGCCGATTATCTCGTGGACTGGTTCAGGCGCAGTGGCGAGGTGTTGAATCGCCT |
| **CP011117.1** | *Pseudomonas fluorescens* strain LBUM223 | ATGGATGATCTGGCGGCCTTCGCGGTGCTGATTGAAGCTGGCAGCTTTACCCTGGCCGCCGAGCAACTGGGTTGCAGTAAGGGGCAGCTGTCCAAGCGCATCAGCCAGTTGGAAGCACAGTTTGCCGTGGTGCTGTTGCACCGTACGACACGCAAACTGAGCCTCACCGCCGCCGGCGCG  GCGTTGCTGCCTCAGGCACAAGCGCTGGTGGTGCAGGTGGAGCGCGCGCGCCAGGCATTGGCGCGGCTCAAAGATGATTTGACCGGCCCTGTGCGCATGACCGTTCCGGTCTCGTTGGGTGAGACCTTCTTTGATGGCTTATTACTGGAGTTCTCCAAACAATACCCGCAGGTCCAGGTC  GAACTGGAGCTTAATAACAGTTACCGCGACTTGGCCCGGGATGGTTTTGACTTGGGCGTACGCTCGGGTGCCATCGAACATGAACGCCTGGTGGCCAAGCCTCTGCTGGCGTGGCGCGAGATGACCTGCGCCAGCCCGGCCTATCTGGAACAGCATGGCGAACCACTGACCCCGGCGGAC  CTGGCGACACACATCTGTTTGCTCAACAGTCACTACAGTGGACGCGAGGAATGGTTGTATCACCAGCAACACGAATTGCTGCGGGTACGGGTAAGCGGCACGTTTGCCTCCAACCACTACAACCTGCTGAAAAAAGCCGCGCTGGTGGGCGCCGGTATCGCGCGTCTGCCATCCTACGTA  TTACCGAAGGAACTGGCCGAAGGCCGGTTACGCTGGCTCCTGCGCGACTATCAGACGCGCAGCATGCCGATGTACCTGGTGCACCCCTACCAGGGTGGCCTGCCGCGGCGTACCCAGGTATTGGCCGATTACCTGGTGGACTGGTTCAGGCGCAGCGGCGAGGCGTTGGATCGGCT |
| **CP006931.1** | *Pseudomonas aeruginosa* SCV20265 | GCTGCGCTCGAACCAGTCGAGCAGGTAATCGGCGAGTACCTGGGTGCGCCGCGGCAGGCCGCCCTGGAACGGATGGACGAGGAACATCGGCGTGCTGCGCGTCTGGTAGTCGCGCAGCAACCAGGCCAGGCGGCCGTCGCCCAGTTCGTCGTGGACCATGTATGACGGCAGCCGGGCGAT  GCCGGTGCCGGCCAGCGCGGCCTTCTTCAACAGGCTGTAGTGGTTGCTGGCGAGAAAGCCGGCAACCCGTACCCGCTCCAGCCGATGGTGGCGGTGATAGAGCCACTCCTCGTGGCCGCTGTAGTGCGAGTTCAGCAGGCACTCGCGCTCGGCCAGCTCGGCCGGGCGTTGCGGCTCGCC  GTGTCGCGCCAGGTAGGCGGGACTGGCGCAGGTGATTTCCTGCAGGACGAACAGCGGCCGCGCCACCAGCCGGGCGTCCTGCTCGACCCCAGAGCGAATCGCCAGGTCGAAGCCCTCGCCAACCAGGTCGCGCAGGCCGTTGTACAGGTCCAGCTCGACGCGCAGCAGCGGATGGCGTTC  CTGGAAGTCCTCCAGCAGTGCATCGAACAGGGTCTCGCCCAGCGAGACCGGGAGGGTCACCCGCACCCGTCCTTCCGCTCGCTCCTGCAAGCGCGCCACCGCCTGCCGCGCGCGCTCGGCCTGCACCAGCAAGGCCTGCGCCTCGGGCAGCAGCGCGGCGCCGGCGGCGGTCAGCGAAAG  ACGGCGGGTGGTGCGGTGCAAGAGGGTCACGCCGAGCTTCTGCTCGAGGACGCCAATGCGCTTCGACAGTTGGCCCTTGCTGCAACCGAGACGCTCGGCGGCGCGGGTGAAACTGCCCAGGTCGTGGAGTACGGCGAAGGCGGCGAGATCGTCCA |
| **CP013144.1** | *Pseudomonas aeruginosa* strain Cu1510 | CGAGCGCCGCAGTTTCACCCGCGCCGCCGAGCGCCTCGGTTGCAGCAAGGGCCAACTGTCGAAGCGCATTGGCGTCCTCGAACAGAAGCTCGGCGTGACCCTCTTGCACCGCACCACCCGCCGTCTTTCGCTGACCGCCGCCGGCGCCGCGCTGCTGCCCGAGGCGCAGGCCTTGCTGGT  GCAGGCCGAGCGCGCGCGGCAGGCGGTGGCGCGCCTGCAGGAGCGAGCGGAAGGACGGGTGCGGGTGACCCTCCCGGTCTCGCTGGGCGAGACCCTGTTCGACGCGCTGCTGGAGGACTTCCAGGAACGCCATCCGCTGCTGCGCGTCGAGCTGGACCTGTACAACGGCCTGCGCGACCT  GGTTGGCGAGGGCTTCGACCTGGCGATCCGCTCTGGGGTCGAGCAGGACGCCCGGCTGGTGGCGCGGCCGCTGTTCGTCCTGCAGGAAATCACCTGCGCCAGTCCCGCCTACCTGGCGCGACATGGCGAGCCGCAACGCCCGGCCGAGCTGGCCGAGCGCGAGTGCCTGCTGAACTCGCA  CTACAGCGGCCATGAGGAGTGGCTCTATCACCGCCGCCATCGGCTGGAGCGGGTACGGGTTTCCGGCTTTCTCGCCAGCAACCACTACAGCCTGTTGAAGAAGGCCGCGCTGGCCGGCACCGGCATCGCCCGGCTGCCGTCATACATGGTCCACGACGAACTGGGCGACGGCCGCCTGGC  CTGGTTGCTGCGCGCCTACCAGACGCGCAGCACGCCGATGTTCCTCGTCCATCCGTTCCAGGGCGGCCTGCCGCGGCGCACCCAGGTGCTCGCCGATTACCTGCTCGACTGGTTCGAGCGCAGC |
| **CP003880.1** | *Pseudomonas* sp. UW4 | ATGGATGACCTGGCCGCGTTCGCCGTATTGATCGAAGCGGGAAGTTTCACCCTGGCGGCGCAGCAGCTCGGTTGCAGCAAAGGCCAGCTGTCCAAGCGCATCAGCCAGCTGGAAGCGCAGTTTTCCGTGGTGCTGTTGCAACGTACCACTCGCCGCTTGAGCCTGACCGCCGCTGGTGCT  GCTTTGTTGCCCCAGGCCCAGGCGTTGGTGGTGCAAGTCGAGCGGGCACGCCAGGCCCTGGCGCGACTGAAGGACGACATGGCCGGACCGGTGCGGATGACGGTCCCGGTGTCGCTCGGGGAAACCTTCTTCGATGGCCTGTTGCTGGAGTTCTCGACCCAGTATCCCCAGGTGCAGATC  GAGCTGGAGCTCAACAACAACTACCGCGACCTGTCCCGCGACGGCTTCGACCTGGCGATTCGCTCTGAGGTGGCCCTTGACCAGCGACTGGTGGCGCGCCCGTTGCTGGCGTGGCAGGAACTGACCTGTGCCAGCCCGGCTTATCTGAAGCAATACGGTGAACCACAAACACCCCAGGCG  CTGGCCGAACATCGCTGCCTGCTCAACAGTCATTACAGCGGCCGCGAAGAATGGCTGTATCACCAGCAGCATGAATTGTTGCGGGTGCGGGTGTCGGGGCCGTTCGCCAGCAACCACTACAACCTGTTGAAGAAAGCCGCACTGGTTGGCGCCGGTATTGCGCGCCTGCCCTCCTACTGT  TTGCCAGCGGAGCTGGCGGACGGGCGTTTGCGATGGCTGCTGCGCGACTATCAGACCCGCAGCATGCCGATGTACCTGGTGCATCCGTATCAGGGTGGTTTGCCCAAGCGTACGCAGGTGCTGGCGGATTATTTGATTGGCTGGTTCAAGCGCAGTGGGGAGGCGTTGGA |
| **CP010945.1** | *Pseudomonas fluorescens* NCIMB 11764 | ATGGATGATCTGGCGGCGTTTGCGGTGTTGATCGAAGCGGGCAGTTTCACCCTGGCGGCGCGGCAACTGGGTTGCAGCAAAGGCCAGTTGTCCAAGCGCATCAGCCTGCTGGAGACGCGGTTTTCGGTGGTGCTGTTGCAACGCACCACCCGCCGCTTGAGTCTGACGGCGGCAGGGGCG  GCGCTGTTGCCGCAGGCTCAGGCCTTGGTGGTGCAAGTGGAAAGGGCGCGCCAGGCATTGGCACGGTTGAAGGACGACATGGCCGGGCCGGTGCGGATGACGGTTCCGGTCTCGTTGGGGGAAACCTTCTTCGATGGCTTGTTGCTGGAGTTTTCCCACCAGTACCCCGAAGTGCAGATC  GAACTCGACCTCGACAACAACTACCGTGATTTGTCCCGGGACGGTTTTGATCTGGCGGTGCGTTCCGATGTGGGCAATGACGAGCGTCTGGTGGCGCGGCCGCTGTTGGCCTGGCACGAACTGACCTGCGCCAGCCCGGCCTACCTTGAGCAGCACGGCGAACCGCTGACGCCGCAGGCA  CTCGCCGAACATCAGTGTTTACTCAACAGTCACTACAGCGGCCGTGAAGAATGGTTGTATCACCAGCAGCACGAGTTATTGCGCGTGCGGGTGTCGGGACCGTTCGCCAGCAACTACTACAGCCTGTTGAAGAAAGCCGCATTGGGTGGCGCGGGTATTGCGCGTCTGCCGTCCTACCTG  CTGCAAAAGGAATTGGCTGACGGGCGTTTGCGCTGGCTCCTGCGTGACTATCAGACTCGTCGCATGCCAATGTACCTGGTGCATCCGTATCAGGGTGGGTTGCCCAAGCGCACGCAAGTGCTGGCGGATTATTTGATTGGGTGGTTCAAGCGCAGTGGGGAGGCACTGGA |
| **CP012676.1** | *Pseudomonas psychrophila* strain L10.10 | ATGGATGACCTGGCGGCGTTCGCAGTGTTGATCGATGCCGGTAGTTTTACGTTGGCGGCGCAGCAGTTGGGCTGTAGCAAGGGGCAGTTGTCCAAACGTATCAGCCAGCTTGAAGCCCAGTTTTCGGTGGTGTTGCTGCACCGCACCACCAGGCGCCTGAGCTTGACCGCCGCCGGTTCT  GCGTTATTGCCACAAGCCCGGGCGCTGGTGATACAGGTGGAGCATGCACGTCAGGCGCTGGCCCGGCTCAAGGACGACATGGCGGGGCCGGTGCGCATGACCGTACCCGTGTCGCTGGGCGAAACGTTTATTGATGCACTGCTGCTGGAGTTCTCCCGCGATTACCCGCAGGTGCAGATT  GAGCTTGAACTCAATAACAGCTATCGCGATATGGCCCGGGACGGCTTTGACCTGGCGATCCGCTCACAAGTGGGCAATCACGAGCGGCTGGTGGCCAAGCCCGTTCTGAACTGGCACGAGATCACTTGCGCCAGCCCTGCCTACCTTGAGCAACACGGCGAGCCGAGTACGCCGCAGCAA  TTGGTCGAGCATCAGTGTTTGCTCAACAGTCATTACAGTGGTCGCGAGGAGTGGCTGTACCACCGCCAGCATGAGCTGCATCGGGTGCGGGTATCGGGGCCGTTTGCCAGTAATCATTACAGTCTGCTGAAAAAGGCCGCCGTGATAGGTGCGGGAATCGCCCGTCTGCCTTCTTACATG  CTCCAGGCCGAACTGGCGGATGGGCGTTTACGCGGGCTGCTGCGCGATTATCAAACCCGAAGCAATCCGATGTATCTGGTGCATCCTTATCAGGGCGGGCTGCCCAGACGCACGCAGGTTCTGGCCGATTATCTAGTGGGCTGGTTCCGGCGTAGCGGTGAGGC |
| **CP009048.1** | *Pseudomonas alkylphenolia* strain KL28 | TGGATGATCTGGCGGCCTTCGCCGTGTTGATGGATGTCGGCAGCTTCACCCAGGCCGCGCAACAACTGGGGTGGAGCAAGGGGCAGCTGTCCAAGCGCATCAGTGCTTTGGAGGCCAGTCATTCGGTAAAGCTGCTGCACCGCACCACGCGGCGTCTGAGTTTGACCGCAGCGGGTGCCA  TGTTGCTGCCCCAGGCCCAGGCATTGGTGCGGCAAATGGAGGGGGCGCGTCAGACTTTGGCCATGCTCAAGGACGAACTGGCTGGCCCGGTGCGCATCACCGTACCGGTGTCGCTAGGCGAGACCTTCTTCGAAGGCTTGCTGCTGGAGTTCGCCGTCGACTATCCGGATCTGCAGGTGG  AACTGGAGCTGAACAATGGCTACCGGGATCTGTTGGCGGAGGGCTTCGATCTGGCGATTCGCACCGAGGTCCAGGACGATGCACGATTGGTTGCGCGCCCGCTGTTGGCCATGCAGGAACTGACCTGCGCCAGCCCTGCCTATCTGCAGCGCCAGGGCGAACCGCACATGCCAGCTGAAT  TGAGCGCTCATCGCTGTTTGCTCAACAGCCACTACAGTGGGCGGGAAGAATGGTTGTATCACCACCAGCATGAGCTGCTGCGGGTGCAGGTCGCCGGCAGCTTTGCCAGCAATCACTACAGCCTGCTGAAGAAAGCTGCATTGCTCGGCACCGGCATCGCCAGGCTACCTTCCTACATGG  TGCATGCGGAGCTTGCCGACGGCCGCTTGCAGTGGTTGCTGCGTGACTACCAGACCCGCAGTGTGCCGTTGTTTCTGGTGCATCCGTATCAGGGCCGGATGCCCAGAAGAGTCCAGGTACTGGCCGACTACCTGCTGCGCTGGTTCAAGCGCAGCAGCCAGGCGTTG |
| CP012639.1 | *Serratia marcescens* strain RSC-14, | AACGACTTGATTCTGTTTGCCCTGATTGTCGACTGCGGCTCGTTCAGCAAGGCGGCGGAGAGCGCCGGCATTACCAGTTCGGTGGTCAGCAAACGCATCGGGCGGTTGGAAAAATCGCTCGGCGCCCGGCTGATGTACCGCACCACCCGCAGCTTGACGCTGACCGAAAGCGGCCAGGCGCTCTACCAGCAGGCCAAGGAGATCGGCGCCAAAGTGCAGGAAGCGCTGTACGCCGTCAGCGAAAAGAGCGAAGAGCTGACCGGCACCATTCGTATGTCGGTGCCGACCATTTCGGGTGAG  CTGCTGCTGAGTGAAAGCGTGGCGGAGTTTTGCGCCCAGCACCCCAGCCTGAAGGTCGAGATGCGGCTGGAAAACCGCTTCGTCGATCTGGTGGAAGAAGGCATCGATCTGGCGATCCGTACCGGCACCATGCCGGATTCGAGCCTGATTGCGCGGCCGATCTTCGATTCCCGCTGGGTGATCGTCTGCTCGCCGGGCTATCTGGAGAACCACCCGGAGCCGCGCAATCCCGACGATCTGCTTAGCCACAACTGCCTGACCTATACCTATCAAGAGAGCGGCACCGCCAACTGGCTGATGAAGCGGCCAGGGCGCAACGAAATCTACGAGCTGCAGGTTGACGGCAACCTGTCGGCCAACAATGCGCGGGCGATCCGCAAAGCGGTGATCGGCGGCCACGGCATCGCCATGGTGCCGCGCTGCATGGTGTACGAAGATTTGCAGGAAGGCAAACTGGCGGAGATCCTGGCCGGCCACTGCGGCAAGGTATTGGGCATTTACGCCGTCTACCCTTATACCCGCAATCTGCCGTTAAAAACTCGCCTGCTGATCGAACATATCATCGGTTCCTATCAA |
| **AE004091.2** | *Pseudomonas aeruginosa* PAO1 | TGGACGATCTCGCCGCCTTCGCCGTACTCCACGACCTGGGCAGTTTCACCCGCGCCGCCGAGCGTCTCGGTTGCAGCAAGGGCCAACTGTCGAAGCGCATTGGCGTCCTCGAGCAGAAGCTCGGCGTGACCCTCTTGCACCGCACCACCCGCCGTCTTTCGCTGACCGCCGCCGGCGCCG  CGCTGCTGCCCGAGGCGCAGGCCTTGCTGGTGCAGGCCGAGCGCGCGCGGCAGGCTGTGGCGCGCTTGCAGGAGCGAGCGGAAGGACGGGTGCGGGTGACCCTCCCGGTCTCGCTGGGCGAGACCCTGTTCGATGCACTGTTGGAGGACTTCCAGGAACGCCATCCGCTGCTGCGCGTCG  AGCTGGACCTGTACAACGGCCTGCGCGACCTGGTTGGTGAGGGCTTCGACCTGGCGATTCGCTCTGGGGTCGAGCAGGACGCCCGGCTGGTGGCGCGGCCGCTGTTCGTCCTGCAGGAAATCACCTGCGCCAGTCCCGCCTACCTGGCGCGACACGGCGAGCCGCAACGCCCGGCTGAGC  TGGCCGAGCGCGAGTGCCTGCTGAACTCGCACTACAGCGGCCACGAGGAGTGGCTCTATCACCGCCACCATCGGCTGGAGCGGGTACGGGTTGCCGGCTTTCTCGCCAGCAACCACTACAGCCTGTTGAAGAAAGCCGCGCTGGCCGGCACCGGCATCGCCCGGCTGCCGTCATACATGG  TCCACGACGAACTGGGCGACGGCCGCCTGGCCTGGTTGCTGCGCGACTACCAGACGCGCAGCACGCCGATGTTCCTCGTCCATCCGTTCCAGGGCGGCCTGCCGCGGCGCACCCAGGTACTCGCCGATTACCTGCTCGACTGGTTCGAGCGCAGC |
| **AP014628.1** | *Pseudomonas* sp. St29 | ATGGATGACCTGGCGGCGTTTGCCGTGCTGGTGGAGGCGGGCAGCTTTACCCTGGCGGCCCAGCGCCTGGGTTGCAGCAAGGGCCAGTTGTCCAAGCGCCTCAGCGCCCTGGAGGCACGCTACGCCGTGGTGCTGTTGCAACGCACCACCCGGCGCCTGGACCTGACCGCCGCCGGCGCG  GCGTTGCTGCCCCAGGCCCAGGCCCTGGTTGCCCAAGTGGAACGAGCGCATCAGGCCCTGGCGCGGCTCAAGGACGACATGGCCGGGCCCGTGCGCCTGACCGTGCCGGTGTCCTTGGGCGAAACCTTTTTCGACGGCCTGCTGCTGGAGTTCTCCCGGCACTACCCCCAGGTGCAGATC  GAGCTGGACCTGAACAACAGCTACCGCGACCTGGCCCGGGAAGGCTTTGACCTGGCCGTGCGTTCCGAGGTGGCCAGCGACCAGCGCCTGGTGGCCCGGCCGCTGCTGGCCTGGCATGAAATGACCTGCGCCAGCCCGGCTTATCTGGAGCAATTCGGTGAGCCGCAGACTCCCGCGGAG  CTGGCCGAGCATCGCTGCCTGCTCAACAGTCACTACAGTGGGCGGGAGGAGTGGCTCTATCACCAGCGTCACGAGCTGCTGCGGGTGCGGGTCTCCGGGCCCTTTGCCAGCAATCACTACAACCTGCTGAAGAAGGCCGCCCTGGTGGGGGCGGGAATAGCCCGGCTGCCGTCCTATGTG  CTGCACAGCGAGCTGGCCGATGGCCGCTTGCGCTGGCTGTTGCGGGACTACCAGACCCGCAGCATGCCGATGTACCTGGTGCACCCCTATCAGGGCGGCCTGCCCAGGCGCACCCAGGTCCTGGCCGATTACCTGATGGACTGGTTCCGCCGCAGCGGTGAGGCGCTGGATCGTCT |
|  |  |  |
